# Supplementary material for: Construction of N−E bonds via Lewis acid-promoted functionalization of chromium-dinitrogen complexes
Source: Nat Commun. 2025 Jan 15;16:674. doi: 10.1038/s41467-025-55998-5 (PMC11733176; doi:10.1038/s41467-025-55998-5)
Supplement: Supplementary file 1 — Supplementary Information [file 41467_2025_55998_MOESM1_ESM.pdf]

Supplementary Information

Manuscript Title:

Construction of N−E bonds via Lewis acid-promoted functionalization of chromium-dinitrogen complexes

Authors:

Zhu-Bao Yin[1], Gao-Xiang Wang[1], Xuechao Yan[1], Junnian Wei[1]* & Zhenfeng Xi[1]*

Affiliations:

[1]Beijing National Laboratory for Molecular Sciences (BNLMS), Key Laboratory of Bioorganic Chemistry and Molecular Engineering of Ministry of Education, College of Chemistry, Peking University, Beijing 100871, China.

Corresponding author email: jnwei@pku.edu.cn; zfxi@pku.edu.cn

Contents

1) General Methods **S2**

2) Experimental Details **S3**

3) Copies of IR Spectra **S14**

4) Copies of NMR Spectra **S21**

5) Copies of UV-Vis Spectra **S25**

6) X-ray Crystallographic Studies **S33**

7) Computational Details **S49**

8) References **S53**

1) General Methods

Unless otherwise noted, all starting materials were commercially available and were used without further purification. Solvents were purified by the Mbraun SPS-800 Solvent Purification System or the FLEANO Solvent Purification System and dried over fresh Na chips and molecular sieves in a glovebox. All manipulations were carried out under an atmosphere of nitrogen by using standard Schlenk techniques or glovebox techniques. The nitrogen in the glovebox was constantly circulated through a copper/molecular sieves catalyst unit. The oxygen and moisture concentrations in the glovebox atmosphere were monitored by an O_2/H_2O Combi-Analyzer to ensure both were always below 0.1 ppm. KC_8 and anhydrous $CrCl_2$ were obtained from Strem; Me_3SiCl, cryptand-222, Et_2AlCl in hexane were obtained from J&K, and Me_3SiCl was dried using molecular sieves; $BF_3{\bullet}Et_2O$, MeOTf, Me_3GeCl and $^{i}Pr_2PCl$ were obtained from TCI. BEt_3 in THF was obtained from Acros.

Organometallic samples for NMR spectroscopic measurements were prepared in a glovebox by the use of J. Young valve NMR tubes (Wilmad 528-JY). ^{1}H, ^{11}B, and ^{15}N NMR spectra were recorded on a Bruker ARX400 spectrometer, Bruker AVANCE III 500 or a Bruker Avance 600 MHz spectrometer at room temperature, unless otherwise noted. All chemical shifts were reported in units of ppm with references to the residual protons of the deuterated solvents for proton chemical shifts. ^{11}B chemical shifts are reported in ppm relative to $BF_3{\bullet}Et_2O$. ^{15}N chemical shifts are reported in ppm relative to liquid NH_3 at 0 ppm. Magnetic moments were measured using the method originally described by Evans, with stock and experimental solutions containing a known amount of a TMSOTMS standard.[1] Elemental analyses were tested on a Vario EL elemental analyzer at the Analytical Center of Peking University. Infrared Spectra of solid samples were recorded on a Bruker AlphaII using a KBr pellet, and Infrared Spectra of samples in solution were recorded using a KBr cell. UV−vis spectra were recorded with an Olis 8453 or Cary 60 spectrophotometer equipped with a cryostat from Unisoku Scientific Instruments, Osaka, Japan.

Compounds **1-crypt**,[2] **^{15}N-1-crypt**,[2] and **5**[3] were synthesized based on the reported procedures.

2) Experimental Details

Preparation of 1-crypt[2]

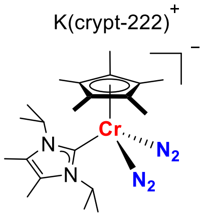

1-crypt

Under an atmosphere of nitrogen, the suspension of excess KC_8 (0.3 mmol, 40.5 mg) in THF (2 mL) was added into the mixture of complex **Cp*(I^{i}Pr$_2$Me$_2$)CrCl** (0.1 mmol, 40.3 mg) and cryptand-222 (0.1 mmol, 37.6 mg) in THF (4 mL). The solution was stirred for 24 h at room temperature and the color turned to orange red gradually. The solvent was removed under reduced pressure and the residues were extracted by THF. The solvent was evaporated under vacuum, leaving orange residues. The residues were washed with cold THF and dried under vacuum to give **1-crypt** as orange powder (65.4 mg, 78%). ^{1}H NMR (400 MHz, THF-d^8) δ 1.48 (s, 21H, C_5Me_5 & $CH(\underline{CH_3})_2$), 1.73 (s, 6H, overlap with THF, $CH(\underline{CH_3})_2$), 2.13 (s, 6H, $\underline{Me}C{=}C\underline{Me}$), 2.55 (t, J = 4.0 Hz, 12H, $N\underline{CH_2}CH_2O$), 3.54 (t, J = 4.0 Hz, 12H, $NCH_2\underline{CH_2}O$), 3.58 (s, 12H, overlap with THF, $O\underline{CH_2}\underline{CH_2}O$), 5.43 – 5.60 (m, 2H, $\underline{CH}(CH_3)_2$). ^{13}C NMR (151 MHz, THF-d^8) δ 9.7 (C_5Me_5), 9.8 ($\underline{Me}C{=}C\underline{Me}$), 23.7 ($CH(\underline{CH_3})_2$), 25.4 ($CH(\underline{CH_3})_2$), 50.7 ($\underline{CH}(CH_3)_2$), 54.3 ($N\underline{CH_2}CH_2O$), 67.9 ($NCH_2\underline{CH_2}O$), 70.8 ($O\underline{CH_2}\underline{CH_2}O$), 86.3 ($\underline{C_5}Me_5$), 123.0 ($Me\underline{C}{=}\underline{C}Me$), 229.6 (Cr-C). IR (KBr, cm^{-1}) $\upsilon(N_2)$: 1869, 1781. The spectral data matched with those reported in the literature.[2]

Preparation of ^{15}*N-1-crypt*[2]

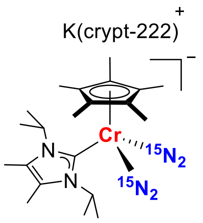

^{15}N-1-crypt

To a 25 mL Schlenk tube containing a magnetic stirring bar were added complex **Cp*(I^{i}Pr$_2$Me$_2$)CrCl** (0.2 mmol, 80.6 mg), cryptand-222 (0.2 mmol, 75.3 mg) and KC_8 (0.6 mmol, 81.0 mg) and THF (8 mL). The mixture was frozen and degassed three times. Then the Schlenk tube was back-filled with $^{15}N_2$ while the mixture was still frozen. The tube was sealed and the solution was stirred at room temperature for 24 h. The solvent was removed under reduced pressure and the residues were extracted by THF. The solvent was evaporated under vacuum, leaving orange residues. The residues were washed with cold THF and dried under vacuum to give **^{15}N-1-crypt** as orange powder (129.5 mg, 77%). ^{15}N NMR (61 MHz, THF-d^8) δ 403.73 (d, $^1J_{NN}$ = 10.8 Hz), 412.78 (d, $^1J_{NN}$ = 10.8 Hz). IR (KBr, cm^{-1}) $\upsilon(^{15}N_2)$: 1807, 1722. The spectral data matched with those reported in the literature.[2]

Preparation of 5[3]

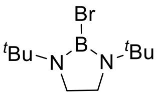

5

A solution of BBr_3 (1.25 g, 5.0 mmol) in 3 mL of hexane was slowly added to the trimethylamine (1.01 g, 10.0 mmol) in 100 mL of hexane at 0 °C, whereby a white precipitate was formed. After warming to room temperature the solution

of *N*, *N′*-di-*tert*-butylethylenediamine (0.86 g, 5.0 mmol) in 10 mL of hexane was added, and the mixture was heated under reflux for 3 h. Then it was filtered, and the filtrate was evaporated to dryness to afford pure **5** as a microcrystalline colorless solid, yield 1.00 g (77%). ^{1}H NMR (400 MHz, C_6D_6) δ 1.30 (s, 18H, ^{t}Bu), 2.92 (s, 4H, CH_2). The spectral data matched with those reported in the literature.[3]

Preparation of 2a

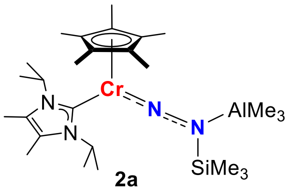

2a

Method A: Under an atmosphere of nitrogen, excess KC_8 (0.3 mmol, 40.5 mg) was added into the THF (6 mL) solution of complex **Cp*Cr(^{i}IPr$_2$Me$_2$)Cl** (0.1 mmol, 40.3 mg). The solution was stirred for 24 h at room temperature, generating the Cr(0)-N_2 complex **1-K**, as evidenced by two peaks (1760 cm^{-1}, 1846 cm^{-1}) in the IR spectra. The solvent was filtered, and the filtrate was concentrated to approximately 4 mL before adding 2 mL of Et_2O. The solution was frozen in the coldwell chilled externally with liquid nitrogen. Meanwhile, a solution of $AlMe_3$ (1 M in hexane, 0.1 mmol, 100 µL) in hexane was also frozen in the coldwell chilled externally with liquid nitrogen. Immediately upon thawing, the solution of $AlMe_3$ was added to the frozen **1-K** equipped with a magnetic stirring bar. The solution was slowly warmed to -70 °C while stirring for 30 min. Then the reaction solution was frozen in the coldwell chilled externally with liquid nitrogen again. Meanwhile, a solution of Me_3SiCl (0.1 mmol, 13 µL) in THF was also frozen in the coldwell chilled externally with liquid nitrogen. Immediately upon thawing, the solution of Me_3SiCl was added to the frozen reaction solution. The solution was slowly warmed to -30 °C while stirring for 70 min, leading to a color change from brown-green to dark green. Volatile materials were removed under vacuum. The solid residues were extracted with hexane/Et_2O. The filtrate was concentrated and placed in a -30 °C freezer, yielding dark green crystals of **2a** (21.6 mg, 40%).

Method B: Under an atmosphere of nitrogen, excess KC_8 (0.3 mmol, 40.5 mg) was added into the THF (6 mL) solution of complex **Cp*Cr(^{i}IPr$_2$Me$_2$)Cl** (0.1 mmol, 40.3 mg). The solution was stirred for 24 h at room temperature, generating the Cr(0)-N_2 complex **1-K**, as evidenced by two peaks (1760 cm^{-1}, 1846 cm^{-1}) in the IR spectra. The solvent was filtered, and the filtrate was concentrated to approximately 4 mL before adding 2 mL of Et_2O. The solution was frozen in the coldwell chilled externally with liquid nitrogen. Meanwhile, a solution of Me_3SiCl (0.1 mmol, 13 µL) in THF was also frozen in the coldwell chilled externally with liquid nitrogen. Immediately upon thawing, the solution of Me_3SiCl was added to the frozen **1-K** equipped with a magnetic stirring bar. The solution was slowly warmed to -70 °C while stirring for 30 min.

Then the reaction solution was frozen in the coldwell chilled externally with liquid nitrogen again. Meanwhile, a solution of $AlMe_3$ (1 M in hexane, 0.1 mmol, 100 μL) in hexane was also frozen in the coldwell chilled externally with liquid nitrogen. Immediately upon thawing, the solution of $AlMe_3$ was added to the frozen reaction solution. The solution was slowly warmed to -30 °C while stirring for 70 min. Volatile materials were removed under vacuum. The solid residues were extracted with hexane/Et_2O. The filtrate was concentrated and placed in a -30 °C freezer, yielding dark green crystals of **2a** (33.5 mg, 62%).

^{1}H NMR (400 MHz, C_6D_6) δ -0.07, 0.56 (br), 0.83, 0.85, 0.87, 0.90, 0.95, 0.97, 1.04, 1.06, 1.51, 6.63 (br), 6.96, 7.36, 32.61 (br). IR (KBr, cm^{-1}) 2980, 2909, 1459, 1366, 1239, 1162, 841, 760, 483. UV-Vis (THF): 588 nm. Anal. Calcd (%). For $C_{27}H_{53}AlCrSiN_4$: C, 59.96; H, 9.88; N, 10.36. Found: C, 60.22; H, 10.13; N, 9.69. μ_{eff} (THF-d^8, Evans method, 25 °C): 2.4(1) μ_B.

Preparation of 2b

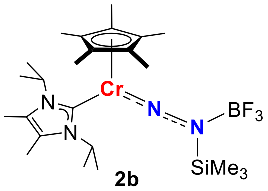

2b

Method A: Under an atmosphere of nitrogen, excess KC_8 (0.3 mmol, 40.5 mg) was added into the THF (6 mL) solution of complex **Cp*Cr(^{i}IPr$_2$Me$_2$)Cl** (0.1 mmol, 40.3 mg). The solution was stirred for 24 h at room temperature, generating the Cr(0)-N_2 complex **1-K**, as evidenced by two peaks (1760 cm^{-1}, 1846 cm^{-1}) in the IR spectra. The solvent was filtered, and the filtrate was concentrated to approximately 4 mL before adding 2 mL of Et_2O. The solution was frozen in the coldwell chilled externally with liquid nitrogen. Meanwhile, a solution of BF_3•Et_2O (0.1 mmol, 12 μL) in Et_2O was also frozen in the coldwell chilled externally with liquid nitrogen. Immediately upon thawing, the solution of BF_3•Et_2O was added to the frozen **1-K** equipped with a magnetic stirring bar. The solution was slowly warmed to -70 °C while stirring for 30 min. Then the reaction solution was frozen in the coldwell chilled externally with liquid nitrogen again. Meanwhile, a solution of Me_3SiCl (0.1 mmol, 13 μL) in THF was also frozen in the coldwell chilled externally with liquid nitrogen. Immediately upon thawing, the solution of Me_3SiCl was added to the frozen reaction solution. The solution was slowly warmed to -30 °C while stirring for 70 min, leading to a color change from brown-green to green. Volatile materials were removed under vacuum. The solid residues were washed with hexane/Et_2O and extracted with Et_2O/THF. The filtrate was concentrated and placed in a -30 °C freezer or room temperature, yielding green crystals of **2b** (30.6 mg, 57%).

Method B: Under an atmosphere of nitrogen, excess KC_8 (0.3 mmol, 40.5 mg) was added into the THF (6 mL) solution of complex **Cp*Cr(^{i}IPr$_2$Me$_2$)Cl** (0.1 mmol, 40.3 mg). The solution was stirred for 24 h at room temperature, generating the Cr(0)-N_2 complex **1-K**, as evidenced

by two peaks (1760 cm^{-1}, 1846 cm^{-1}) in the IR spectra. The solvent was filtered, and the filtrate was concentrated to approximately 4 mL before adding 2 mL of Et_2O. The solution was frozen in the coldwell chilled externally with liquid nitrogen. Meanwhile, a solution of Me_3SiCl (0.1 mmol, 13 µL) in THF was also frozen in the coldwell chilled externally with liquid nitrogen. Immediately upon thawing, the solution of Me_3SiCl was added to the frozen **1-K** equipped with a magnetic stirring bar. The solution was slowly warmed to -70 °C while stirring for 30 min. Then the reaction solution was frozen in the coldwell chilled externally with liquid nitrogen again. Meanwhile, a solution of BF_3•Et_2O (0.1 mmol, 12 µL) in Et_2O was also frozen in the coldwell chilled externally with liquid nitrogen. Immediately upon thawing, the solution of BF_3•Et_2O was added to the frozen reaction solution. The solution was slowly warmed to -30 °C while stirring for 70 min. Volatile materials were removed under vacuum. The solid residues were washed with hexane/Et_2O and extracted with Et_2O/THF. The filtrate was concentrated and placed in a -30 °C freezer or room temperature, yielding green crystals of **2b** (38.1 mg, 71%). ^1H NMR (400 MHz, THF-d^8) δ -0.12 (br), 0.07, 0.19, 0.20, 2.04, 2.28, 6.98, 14.96 (br), 25.29 (br), 32.25(br). IR (KBr, cm^{-1}) 2981, 2917, 1461, 1381, 1145, 1098, 1050, 940, 893, 840, 826. UV-Vis (THF): 611 nm. Anal. Calcd (%). For $C_{24}H_{44}BCrF_3SiN_4$: C, 53.73; H, 8.27; N, 10.44. Found: C, 54.29; H, 8.32; N, 9.33. μ_{eff} (THF-d^8, Evans method, 25 °C): 2.7(1) μ_B.

Preparation of 3

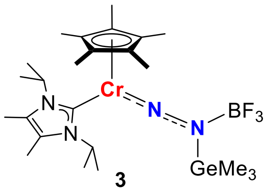

3

Method A: Under an atmosphere of nitrogen, excess KC_8 (0.3 mmol, 40.5 mg) was added into the THF (6 mL) solution of complex **Cp*Cr(^{i}IPr$_2$Me$_2$)Cl** (0.1 mmol, 40.3 mg). The solution was stirred for 24 h at room temperature, generating the Cr(0)-N_2 complex **1-K**, as evidenced by two peaks (1760 cm^{-1}, 1846 cm^{-1}) in the IR spectra. The solvent was filtered, and the filtrate was concentrated to approximately 4 mL before adding 2 mL of Et_2O. The solution was frozen in the coldwell chilled externally with liquid nitrogen. Meanwhile, a solution of BF_3•Et_2O (0.1 mmol, 12 µL) in Et_2O was also frozen in the coldwell chilled externally with liquid nitrogen. Immediately upon thawing, the solution of BF_3•Et_2O was added to the frozen **1-K** equipped with a magnetic stirring bar. The solution was slowly warmed to -70 °C while stirring for 30 min. Then the reaction solution was frozen in the coldwell chilled externally with liquid nitrogen again. Meanwhile, a solution of Me_3GeCl (0.1 mmol, 13 µL) in THF was also frozen in the coldwell chilled externally with liquid nitrogen. Immediately upon thawing, the solution of Me_3GeCl was added to the frozen reaction solution. The solution was slowly warmed to -30 °C while stirring for 70 min, leading to a color change from brown-green to

green. Volatile materials were removed under vacuum. The solid residues were washed with hexane/Et_2O and extracted with Et_2O/THF. The filtrate was concentrated and placed in a -30 °C freezer or room temperature, yielding green crystals of **3** (29.1 mg, 50%).

Method B: Under an atmosphere of nitrogen, excess KC_8 (0.3 mmol, 40.5 mg) was added into the THF (6 mL) solution of complex **Cp*Cr(^{i}IPr$_2$Me$_2$)Cl** (0.1 mmol, 40.3 mg). The solution was stirred for 24 h at room temperature, generating the Cr(0)-N_2 complex **1-K**, as evidenced by two peaks (1760 cm^{-1}, 1846 cm^{-1}) in the IR spectra. The solvent was filtered, and the filtrate was concentrated to approximately 4 mL before adding 2 mL of Et_2O. The solution was frozen in the coldwell chilled externally with liquid nitrogen. Meanwhile, a solution of Me_3GeCl (0.1 mmol, 13 µL) in THF was also frozen in the coldwell chilled externally with liquid nitrogen. Immediately upon thawing, the solution of Me_3GeCl was added to the frozen **1-K** equipped with a magnetic stirring bar. The solution was slowly warmed to -70 °C while stirring for 30 min. Then the reaction solution was frozen in the coldwell chilled externally with liquid nitrogen again. Meanwhile, a solution of BF_3•Et_2O (0.1 mmol, 12 µL) in Et_2O was also frozen in the coldwell chilled externally with liquid nitrogen. Immediately upon thawing, the solution of BF_3•Et_2O was added to the frozen reaction solution. The solution was slowly warmed to -30 °C while stirring for 70 min. Volatile materials were removed under vacuum. The solid residues were washed with hexane/Et_2O and extracted with Et_2O/THF. The filtrate was concentrated and placed in a -30 °C freezer or room temperature, yielding green crystals of **3** (35.5 mg, 61%).

^{1}H NMR (400 MHz, THF-d^8) δ 0.98 (br), 1.56, 1.89, 2.29, 3.76, 6.66, 15.12(br), 25.99 (br), 34.23 (br). IR (KBr, cm^{-1}) 2978, 2938, 2911, 1459, 1415, 1383, 1364, 1104, 1090, 1050, 939, 833, 765. UV-Vis (THF): 626 nm. Anal. Calcd (%). For $C_{24}H_{44}BCrF_3GeN_4$: C, 49.61; H, 7.63; N, 9.64. Found: C, 50.42; H, 8.05; N, 9.26. μ_{eff} (THF-d^8, Evans method, 25 °C): 2.4(1) μ_B.

Preparation of 4

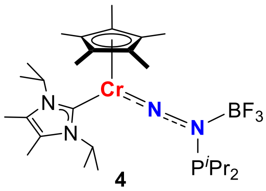

4

Method A: Under an atmosphere of nitrogen, excess KC_8 (0.3 mmol, 40.5 mg) was added into the THF (6 mL) solution of complex **Cp*Cr(^{i}IPr$_2$Me$_2$)Cl** (0.1 mmol, 40.3 mg). The solution was stirred for 24 h at room temperature, generating the Cr(0)-N_2 complex **1-K**, as evidenced by two peaks (1760 cm^{-1}, 1846 cm^{-1}) in the IR spectra. The solvent was filtered, and the filtrate was concentrated to approximately 4 mL before adding 2 mL of Et_2O. The solution was frozen in the coldwell chilled externally with liquid nitrogen. Meanwhile, a solution of BF_3•Et_2O (0.1 mmol, 12 µL) in Et_2O was also frozen in the coldwell chilled externally with liquid nitrogen. Immediately upon thawing, the solution of BF_3•Et_2O was added to the frozen

1-K equipped with a magnetic stirring bar. The solution was slowly warmed to -70 °C while stirring for 30 min. Then the reaction solution was frozen in the coldwell chilled externally with liquid nitrogen again. Meanwhile, a solution of $^{i}Pr_2PCl$ (0.1 mmol, 16 µL) in THF was also frozen in the coldwell chilled externally with liquid nitrogen. Immediately upon thawing, the solution of $^{i}Pr_2PCl$ was added to the frozen reaction solution. The solution was slowly warmed to -30 °C while stirring for 70 min, leading to a color change from brown-green to green. Volatile materials were removed under vacuum. The solid residues were washed with hexane/Et_2O and extracted with Et_2O/THF. The filtrate was concentrated and placed in a -30 °C freezer or room temperature, yielding green crystals of **4** (17.4 mg, 30%).

Method B: Under an atmosphere of nitrogen, excess KC_8 (0.3 mmol, 40.5 mg) was added into the THF (6 mL) solution of complex **Cp*Cr(^{i}IPr$_2$Me$_2$)Cl** (0.1 mmol, 40.3 mg). The solution was stirred for 24 h at room temperature, generating the Cr(0)-N_2 complex **1-K**, as evidenced by two peaks (1760 cm^{-1}, 1846 cm^{-1}) in the IR spectra. The solvent was filtered, and the filtrate was concentrated to approximately 4 mL before adding 2 mL of Et_2O. The solution was frozen in the coldwell chilled externally with liquid nitrogen. Meanwhile, a solution of $^{i}Pr_2PCl$ (0.1 mmol, 16 µL) in THF was also frozen in the coldwell chilled externally with liquid nitrogen. Immediately upon thawing, the solution of $^{i}Pr_2PCl$ was added to the frozen **1-K** equipped with a magnetic stirring bar. The solution was slowly warmed to -70 °C while stirring for 30 min. Then the reaction solution was frozen in the coldwell chilled externally with liquid nitrogen again. Meanwhile, a solution of BF_3•Et_2O (0.1 mmol, 12 µL) in Et_2O was also frozen in the coldwell chilled externally with liquid nitrogen. Immediately upon thawing, the solution of BF_3•Et_2O was added to the frozen reaction solution. The solution was slowly warmed to -30 °C while stirring for 70 min. Volatile materials were removed under vacuum. The solid residues were washed with hexane/Et_2O and extracted with Et_2O/THF. The filtrate was concentrated and placed in a -30 °C freezer or room temperature, yielding green crystals of **4** (23.8 mg, 41%).

^{1}H NMR (400 MHz, THF-d^8) δ -6.02, -0.54 (br), 1.56, 1.66, 2.46, 6.15, 15.26 (br), 25.61 (br), 28.91 (br). The ^{31}P NMR could not be detected even at high concentrations, presumably as a consequence of paramagnetic and the presence of the quadrupolar ^{11}B nucleus. IR (KBr, cm^{-1}) 2941, 2863, 1462, 1369, 1099, 1059, 930, 866. UV-Vis (THF): 616 nm. Anal. Calcd (%). For $C_{27}H_{49}BCrF_3N_4P$: C, 55.87; H, 8.51; N, 9.65. Found: C, 55.99; H, 8.98; N, 9.26. μ_{eff} (THF-d^8, Evans method, 25 °C): 2.7(1) μ_B.

Preparation of 7

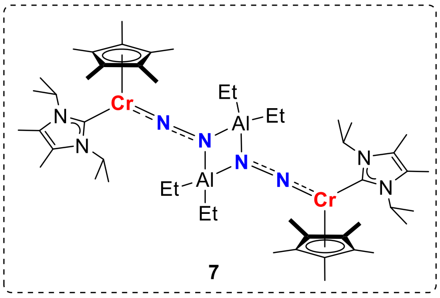

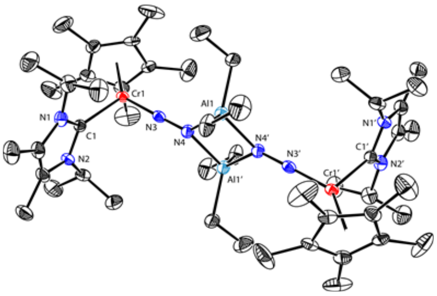

Under an atmosphere of nitrogen, excess KC_8 (0.3 mmol, 40.5 mg) was added into the THF (6 mL) solution of complex **Cp*Cr(^{i}IPr$_2$Me$_2$)Cl** (0.1 mmol, 40.3 mg). The solution was stirred for 24 h at room temperature, generating the Cr(0)-N_2 complex **1-K**, as evidenced by two peaks (1760 cm^{-1}, 1846 cm^{-1}) in the IR spectra. The solvent was filtered, and the filtrate was concentrated to approximately 4 mL before adding 2 mL of Et_2O. The solution was frozen in the coldwell chilled externally with liquid nitrogen. Meanwhile, a solution of Et_2AlCl (1 M in hexane, 0.1 mmol, 100 µL) in hexane was also frozen in the coldwell chilled externally with liquid nitrogen. Immediately upon thawing, the solution of Et_2AlCl was added to the frozen **1-K** equipped with a magnetic stirring bar. The solution was slowly warmed to -30 °C while stirring for 70 min, leading to a color change from brown-green to brown-red. Volatile materials were removed under vacuum. The solid residues were extracted with hexane and filtered. The filtrate was concentrated and placed in a -30 °C freezer, yielding brown-red crystals of **7**, which is too poor quality to be discussed.

Preparation of 6

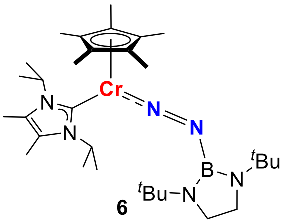

Under an atmosphere of nitrogen, excess KC_8 (0.3 mmol, 40.5 mg) was added into the THF (6 mL) solution of complex **Cp*Cr(^{i}IPr$_2$Me$_2$)Cl** (0.1 mmol, 40.3 mg). The solution was stirred for 24 h at room temperature, generating the Cr(0)-N_2 complex **1-K**, as evidenced by two peaks (1760 cm^{-1}, 1846 cm^{-1}) in the IR spectra. The solvent was filtered, and the filtrate was concentrated to approximately 4 mL before adding 2 mL of Et_2O. The solution was frozen in the coldwell chilled externally with liquid nitrogen. Meanwhile, a solution of **5** (0.1 mmol, 26.1 mg) in Et_2O was also frozen in the coldwell chilled externally with liquid nitrogen. Immediately upon thawing, the solution of **5** was added to the frozen **1-K** equipped with a magnetic stirring bar. The solution was slowly warmed to -30 °C while stirring for 70 min, leading to a color change from brown-green to brown red. Volatile materials were removed under vacuum, the solid residues were extracted with hexane and filtered. The filtrate was concentrated and placed in a -30 °C freezer, yielding brown red crystals

of **6** (16.1 mg, 28%).

^{1}H NMR (400 MHz, C_6D_6) δ -15.98, 0.63 (br), 1.04, 1.05, 1.18, 1.28, 1.31, 1.37, 1.42, 1.49, 1.52, 1.71, 6.96, 7.36, 13.21 (br), 27.90 (br), 46.63 (br). IR (KBr, cm^{-1}) $\upsilon(N_2)$: 1602. UV-Vis (THF): 373, 461, 561 nm. Anal. Calcd (%). For $C_{31}H_{57}BCrN_6$: C, 64.57; H, 9.96; N, 14.57. Found: C, 64.85; H, 10.26; N, 13.77. μ_{eff} (THF-d^8, Evans method, 25 °C): 2.8(1) μ_B.

^{15}N-6 was synthesized using **^{15}N-1-crypt [Cp*Cr(^{i}IPr$_2$Me$_2$)(^{15}N$_2$)$_2$(K(crypt))]** and **5** in a argon atmosphere glovebox. IR (KBr, cm^{-1}) $\upsilon(^{15}N_2)$: 1540.

Preparation of 8

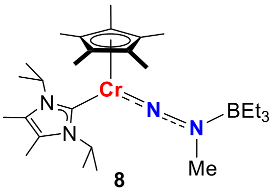

8

Method A: In the glovebox, a THF/Et_2O (4 mL/2 mL) solution of **1-crypt** (0.1 mmol, 84.0 mg) was frozen in the coldwell chilled externally with liquid nitrogen. Meanwhile, a solution of BEt_3 (0.1 mmol, 100 µL) in THF was also frozen in the coldwell chilled externally with liquid nitrogen. Immediately upon thawing, the solution of BEt_3 was added to the frozen **1-crypt** equipped with a magnetic stirring bar. The solution was slowly warmed to -70 °C while stirring for 30 min. Then the reaction solution was frozen in the coldwell chilled externally with liquid nitrogen again. Meanwhile, a solution of MeOTf (0.1 mmol, 12 µL) in Et_2O was also frozen in the coldwell chilled externally with liquid nitrogen. Immediately upon thawing, the solution of MeOTf was added to the frozen reaction solution. The solution was slowly warmed to -30 °C while stirring for 70 min, leading to a color change from orange-red to green. Volatile materials were removed under vacuum. The solid residues were washed with hexane and extracted with Et_2O. The filtrate was concentrated and placed in a -30 °C freezer, yielding green crystals of **8** (27.4 mg, 54%).

Method B: Under an atmosphere of nitrogen, excess KC_8 (0.3 mmol, 40.5 mg) was added into the THF (6 mL) solution of complex **Cp*Cr(^{i}IPr$_2$Me$_2$)Cl** (0.1 mmol, 40.3 mg). The solution was stirred for 24 h at room temperature, generating the Cr(0)-N_2 complex **1-K**, as evidenced by two peaks (1760 cm^{-1}, 1846 cm^{-1}) in the IR spectra. The solvent was filtered, and the filtrate was concentrated to approximately 4 mL before adding 2 mL of Et_2O. The solution was frozen in the coldwell chilled externally with liquid nitrogen. Meanwhile, a solution of BEt_3 (0.1 mmol, 100 µL) in THF was also frozen in the coldwell chilled externally with liquid nitrogen. Immediately upon thawing, the solution of BEt_3 was added to the frozen **1-K** equipped with a magnetic stirring bar. The solution was slowly warmed to -70 °C while stirring for 30 min. Then the reaction solution was frozen in the coldwell chilled externally with liquid nitrogen again. Meanwhile, a solution of MeOTf (0.1 mmol, 12 µL) in Et_2O was also frozen in the

coldwell chilled externally with liquid nitrogen. Immediately upon thawing, the solution of MeOTf was added to the frozen reaction solution. The solution was slowly warmed to -30 °C while stirring for 70 min, leading to a color change from brown-green to green. Volatile materials were removed under vacuum. The solid residues were washed with hexane and extracted with Et_2O. The filtrate was concentrated and placed in a -30 °C freezer, yielding green crystals of **8** (24.4 mg, 48%).

Method C: Under an atmosphere of nitrogen, excess KC_8 (0.3 mmol, 40.5 mg) was added into the THF (6 mL) solution of complex **Cp*Cr(^{i}IPr$_2$Me$_2$)Cl** (0.1 mmol, 40.3 mg). The solution was stirred for 24 h at room temperature, generating the Cr(0)-N_2 complex **1-K**, as evidenced by two peaks (1760 cm^{-1}, 1846 cm^{-1}) in the IR spectra. The solvent was filtered, and the filtrate was concentrated to approximately 4 mL before adding 2 mL of Et_2O. The solution was frozen in the coldwell chilled externally with liquid nitrogen. Meanwhile, a solution of MeOTf (0.1 mmol, 12 µL) in Et_2O was also frozen in the coldwell chilled externally with liquid nitrogen. Immediately upon thawing, the solution of MeOTf was added to the frozen **1-K** equipped with a magnetic stirring bar. The solution was slowly warmed to -70 °C while stirring for 30 min. Then the reaction solution was frozen in the coldwell chilled externally with liquid nitrogen again. Meanwhile, a solution of BEt_3 (0.1 mmol, 100 µL) in THF was also frozen in the coldwell chilled externally with liquid nitrogen. Immediately upon thawing, the solution of BEt_3 was added to the frozen reaction solution. The solution was slowly warmed to -30 °C while stirring for 70 min. Volatile materials were removed under vacuum. The solid residues were extracted with Et_2O. The filtrate was concentrated and placed in a -30 °C freezer, yielding a few crystals of **8** and brown green oil.

Method D: In the glovebox, BEt_3 (1 M in THF, 0.05 mmol, 50 µL) was added to a pre-cooled (-30 °C) THF/Et_2O (3 mL/1.5 mL) solution of **1-crypt** (0.05 mmol, 42.0 mg). The solution was then warmed to room temperature while stirring for 30 min. The reaction solution was cooled to -30 °C again and the pre-cooled (-30 °C) Et_2O solution of MeOTf (0.05 mmol, 6 µL) was added. The solution was then warmed to room temperature while stirring for 40 min, leading to a color change from orange-red to yellow-green. Volatile materials were removed under vacuum. The solid residues were extracted with Et_2O. The filtrate was concentrated and placed in a -30 °C freezer, yielding green crystals of **8** and some unspecified by-products, making it difficult to determine the yield of product **8**.

^{1}H NMR (400 MHz, THF-d^8) δ -2.83 (br), -1.23, 1.41, 1.43, 2.25, 5.09, 15.81 (br), 25.36 (br), 34.00 (br). ^{11}B NMR (160 MHz, THF-d^8) 21.41. IR (KBr, cm^{-1}) 2974, 2930, 2898, 2853, 2811,

1476, 1362, 1295, 1219, 1041, 800. UV-Vis (THF): 342, 463, 654 nm. Anal. Calcd (%). For $C_{28}H_{53}BCrN_4$: C, 66.13; H, 10.50; N, 11.02. Found: C, 66.57; H, 10.66; N, 10.76. μ_{eff} (THF-d^8, Evans method, 25 °C): 3.2(1) μ_B.

Preparation of 9

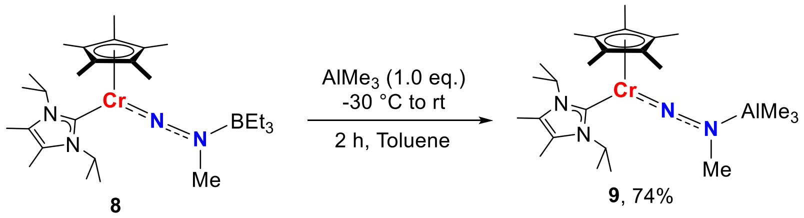

In the glovebox, $AlMe_3$ (1 M in hexane, 0.1 mmol, 100 µL) was added to a pre-cooled (-30 °C) toluene (6 mL) solution of **8** (0.1 mmol, 50.8 mg), and the solution turned to bright green. After the solution was stirred at room temperature for 2 hours, the solvent was removed under reduced pressure, and the residues were extracted by hexane. The solvent was concentrated and placed in a -30 °C freezer, yielding bright green crystals of **9** (35.7 mg, 74%).

^{1}H NMR (400 MHz, C_6D_6) δ -0.10, -0.07, 0.41 (br), 0.87, 0.90, 0.91, 0.95, 0.97, 1.04, 1.05, 1.06, 1.07, 1.28, 1.36, 1.51, 4.87, 6.96, 7.36, 13.75 (br), 27.16 (br), 35.18 (br). IR (KBr, cm^{-1}): 2977, 2913, 2848, 1459, 1361, 1297, 1221, 1165, 686. UV-Vis (THF): 382, 470, 628 nm. Anal. Calcd (%). For $C_{25}H_{47}AlCrN_4$: C, 62.21; H, 9.82; N, 11.61. Found: C, 62.55; H, 9.58; N, 10.28. μ_{eff} (THF-d^8, Evans method, 25 °C): 2.8(1) μ_B.

Preparation of 11

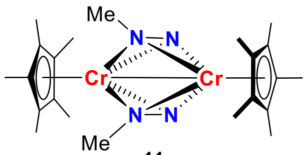

11

In the glovebox, IMe_4 (0.05 mmol, 6.2 mg, IMe_4 = 1,3-dimethyl-4,5-dimethylimidazol-2-ylidene) was added to a pre-cooled (-30 °C) THF/Et_2O (1 mL/1mL) solution of **8** (0.05 mmol, 25.0 mg), and the solution turned to red-brown. After the solution was stirred at room temperature for 4 hours, the solvent was removed under reduced pressure, and the residues were extracted by hexane. The solvent was concentrated and placed in a -30 °C freezer, yielding orange red crystals of **11** and few colorless $I^iPr_2Me_2$ crystals. Although numerous orange-red crystals were obtained, ^{1}H NMR revealed the formation of $I^iPr_2Me_2$ and IMe_4•BEt_3 (IMe_4•BEt_3 can be synthesized independently from IMe_4 and BEt_3 in toluene), making it difficult to determine the exact yield of **11** yet.

The equilibrium between 1-crypt and the Lewis acids Cr–N_2 adducts 10-BEt_3 or 10-$AlMe_3$

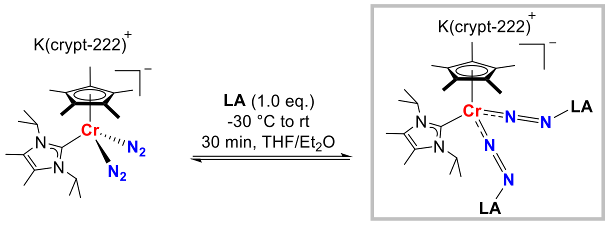

1-crypt or **^{15}N-1-crypt**

10-BEt_3, 10-$AlMe_3$ or **^{15}N-10-BEt_3**

In the glovebox, BEt_3 (1 M in THF, 0.05 mmol, 50 μL or 0.1 mmol, 100 μL) or $AlMe_3$ (1 M in hexane, 0.05 mmol, 50 μL) was added to a pre-cooled (-30 °C) THF/Et_2O (3 mL/1.5 mL) solution of **1-crypt** (0.05 mmol, 42.0 mg). After the solution was stirred at room temperature for 30 min, the IR spectra of the reaction mixture were tested. IR (KBr, cm^{-1}) $\upsilon(N_2)$: 1900, 1869, 1781, 1738 for BEt_3, 1838, 1807, 1722, 1685 for BEt_3 with ^{15}N-labeled **^{15}N-1-crypt**; 1911, 1869, 1781, 1755 for $AlMe_3$.

Addition information for IR analysis: when two substituents exhibit similar vibrational frequencies, coupling of these vibrations occurs, resulting in one shifting toward a higher frequency and the other toward a lower frequency. In our Cr dinitrogen complexes, the presence of two chemically equivalent terminal-coordinated N_2 units leads to vibrational coupling, manifesting as two sets of IR signal peaks. Coordination with a Lewis acid will assist in activating the dinitrogen, further shifting one peak toward lower frequencies and the other toward higher frequencies.

In-situ ^{15}N NMR experiment between 1-crypt and 2.0 equiv. BEt_3: In the argon glovebox, BEt_3 (1 M in THF, 0.02 mmol, 20 μL) was added to a pre-cooled (-30 °C) THF-d^8 (0.5 mL) solution of **1-crypt** (0.01 mmol, 8.4 mg) in a J-young NMR tube. This sample was stored below 0 °C until testing began, with approximately 20 minutes between BEt_3 addition and the start of the test. The test results are shown in Figure S22.

3) Copies of IR Spectra

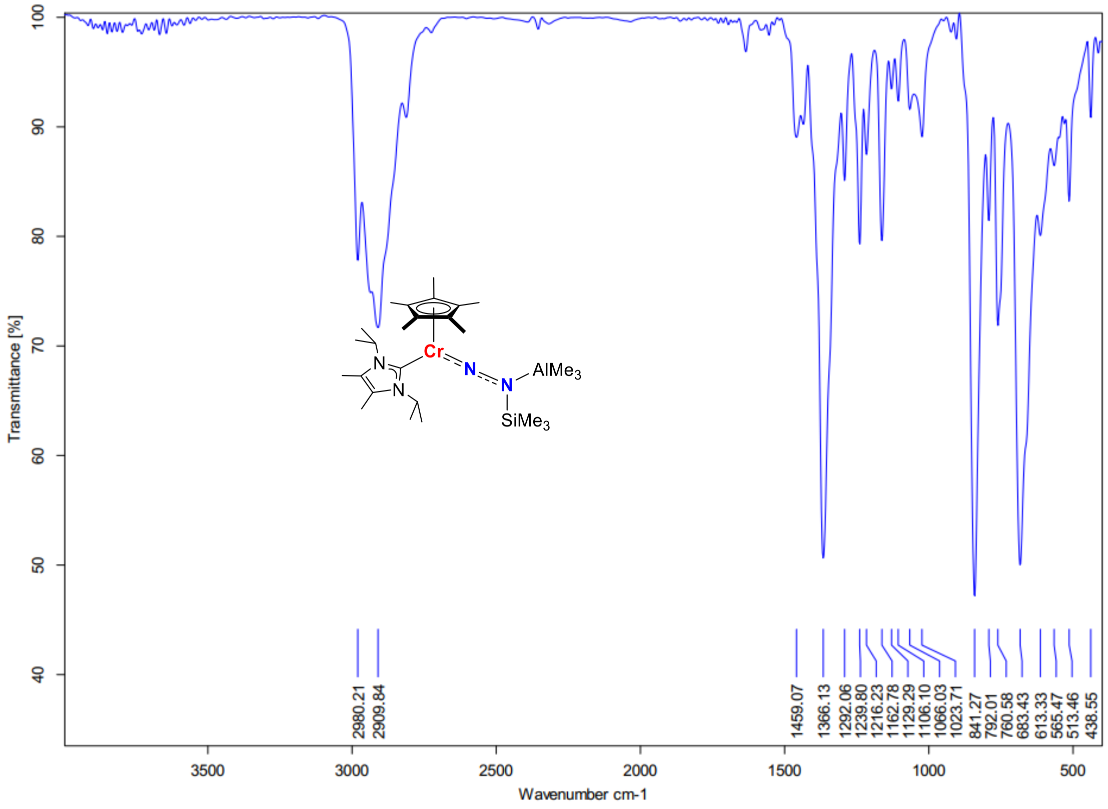

Figure S1 IR spectrum of **2a** in KBr pellet at room temperature

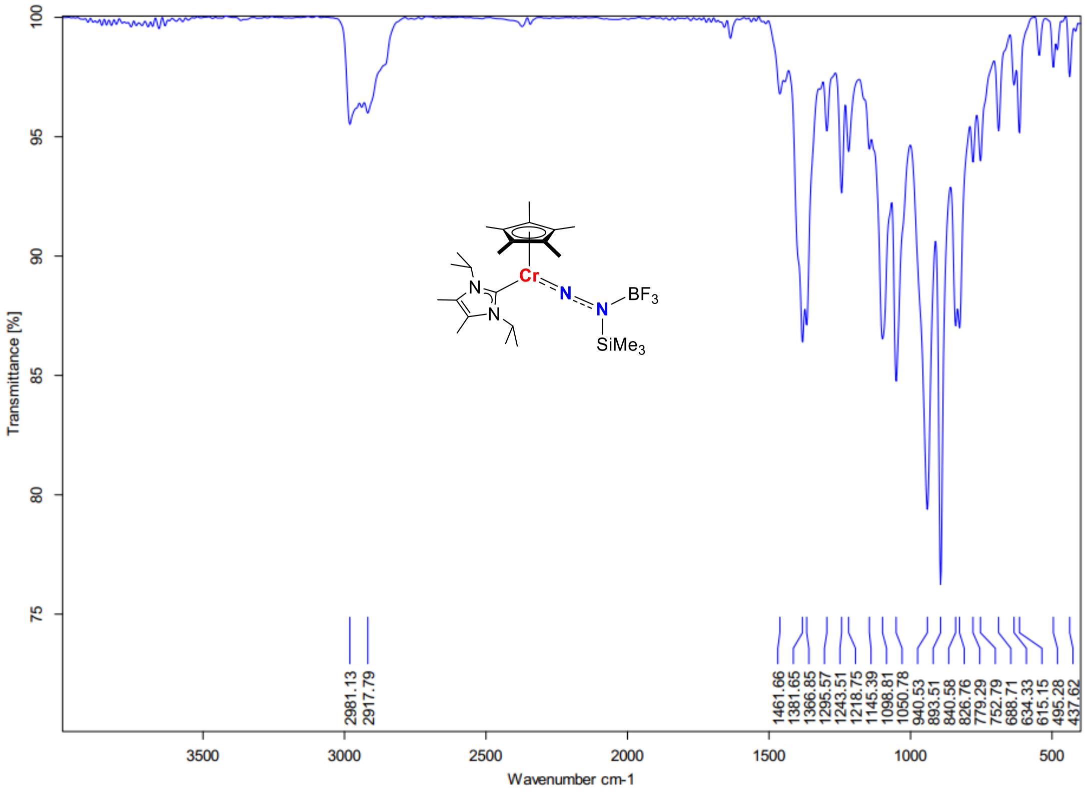

Figure S2 IR spectrum of **2b** in KBr pellet at room temperature

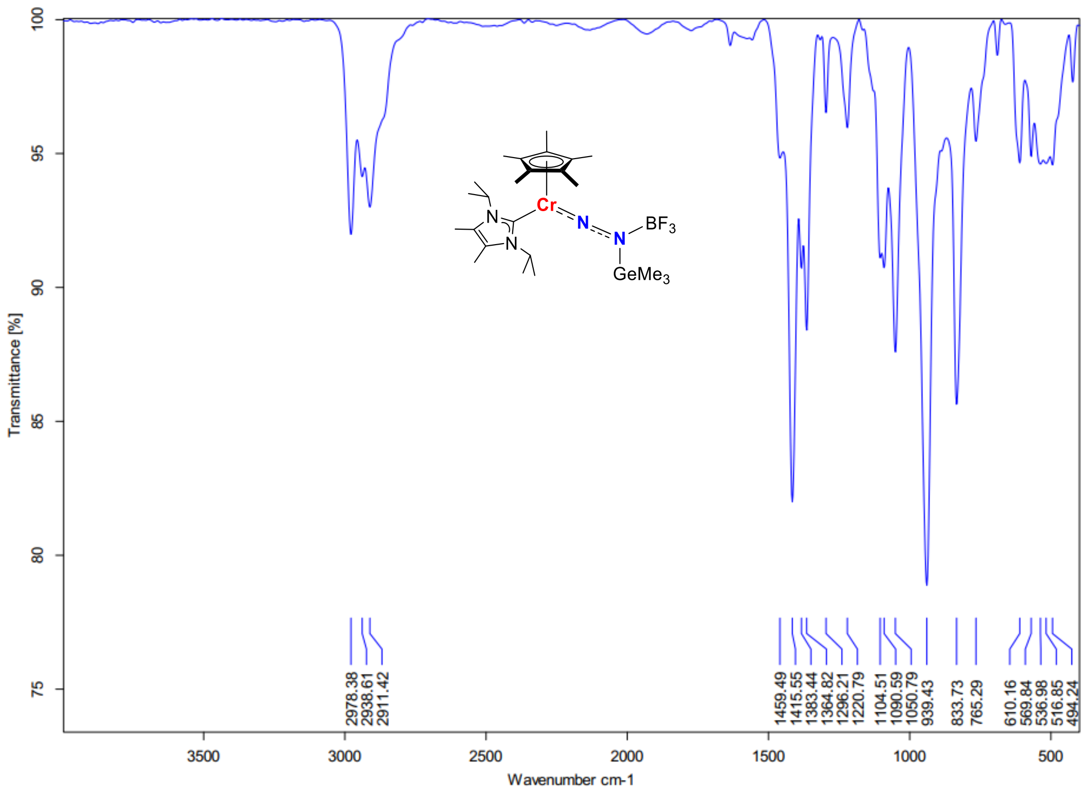

Figure S3 IR spectrum of **3** in KBr pellet at room temperature

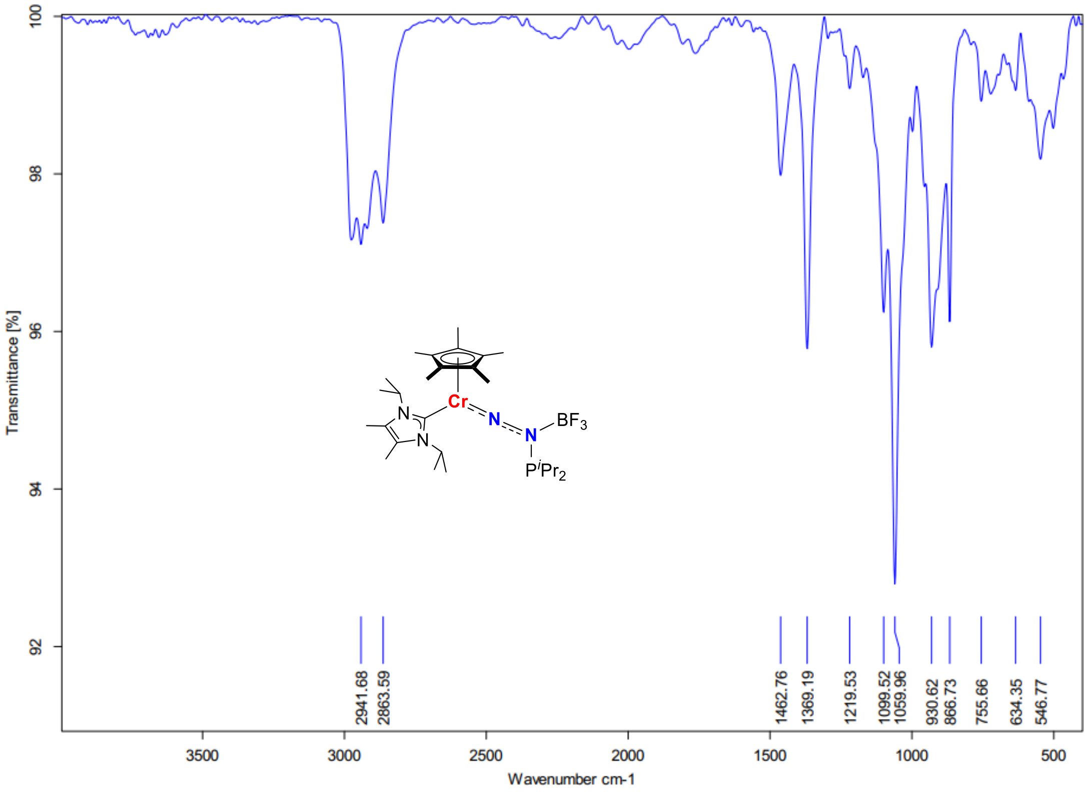

Figure S4 IR spectrum of **4** in KBr pellet at room temperature

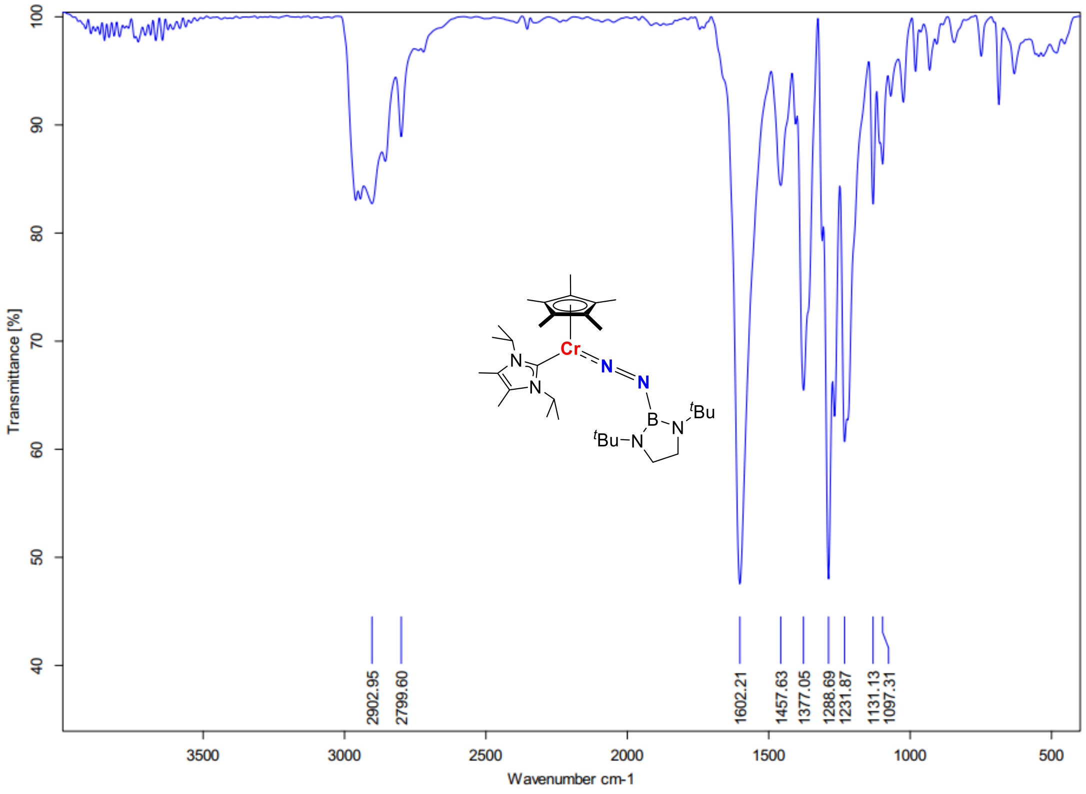

Figure S5 IR spectrum of **6** in KBr pellet at room temperature

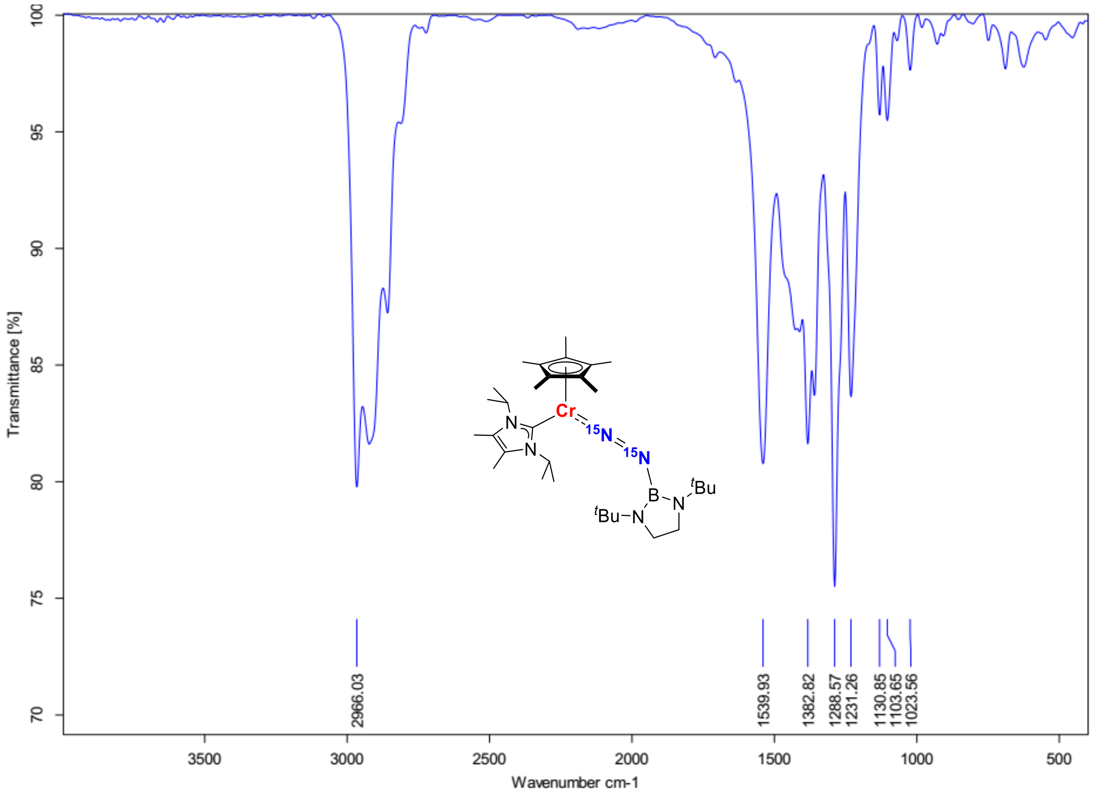

Figure S6 IR spectrum of **^{15}N-6** in KBr pellet at room temperature

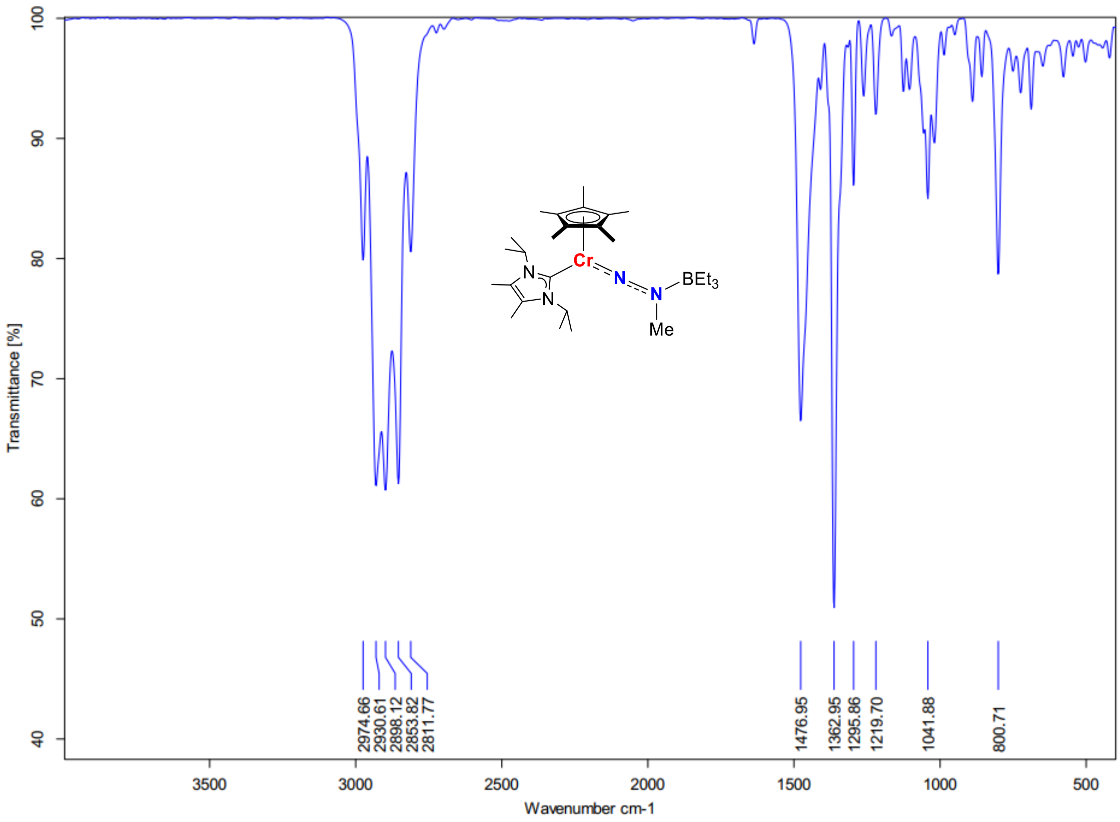

Figure S7 IR spectrum of **8** in KBr pellet at room temperature

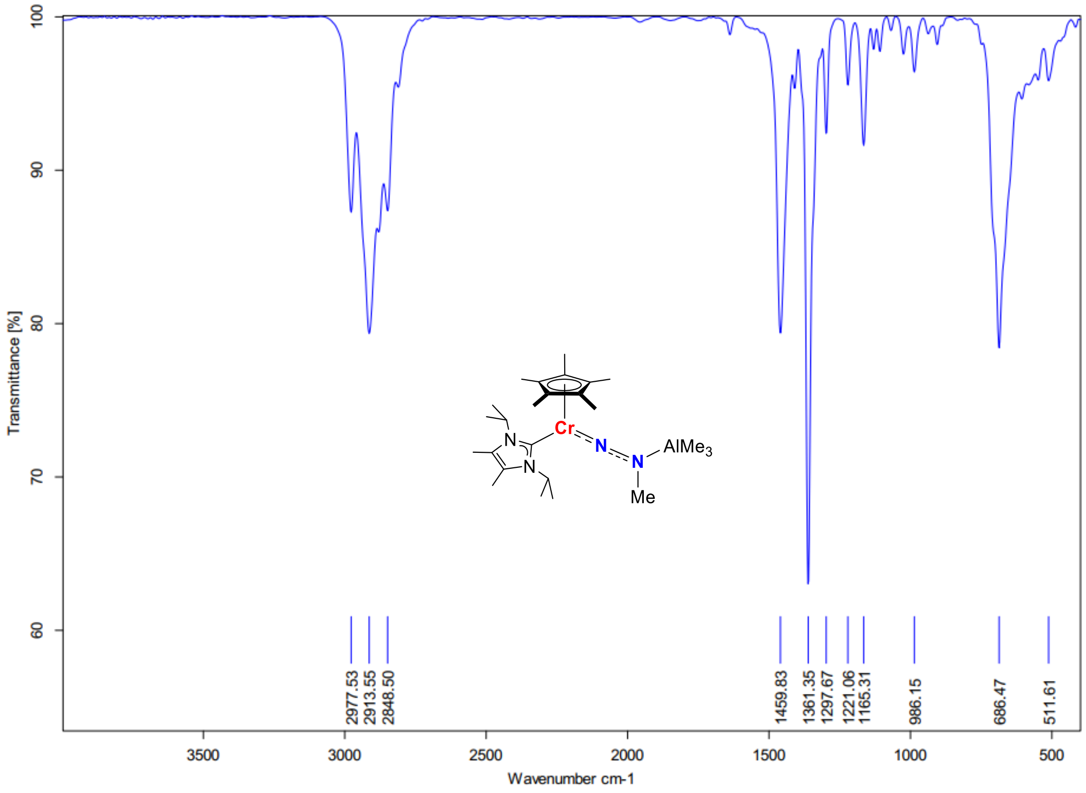

Figure S8 IR spectrum of **9** in KBr pellet at room temperature

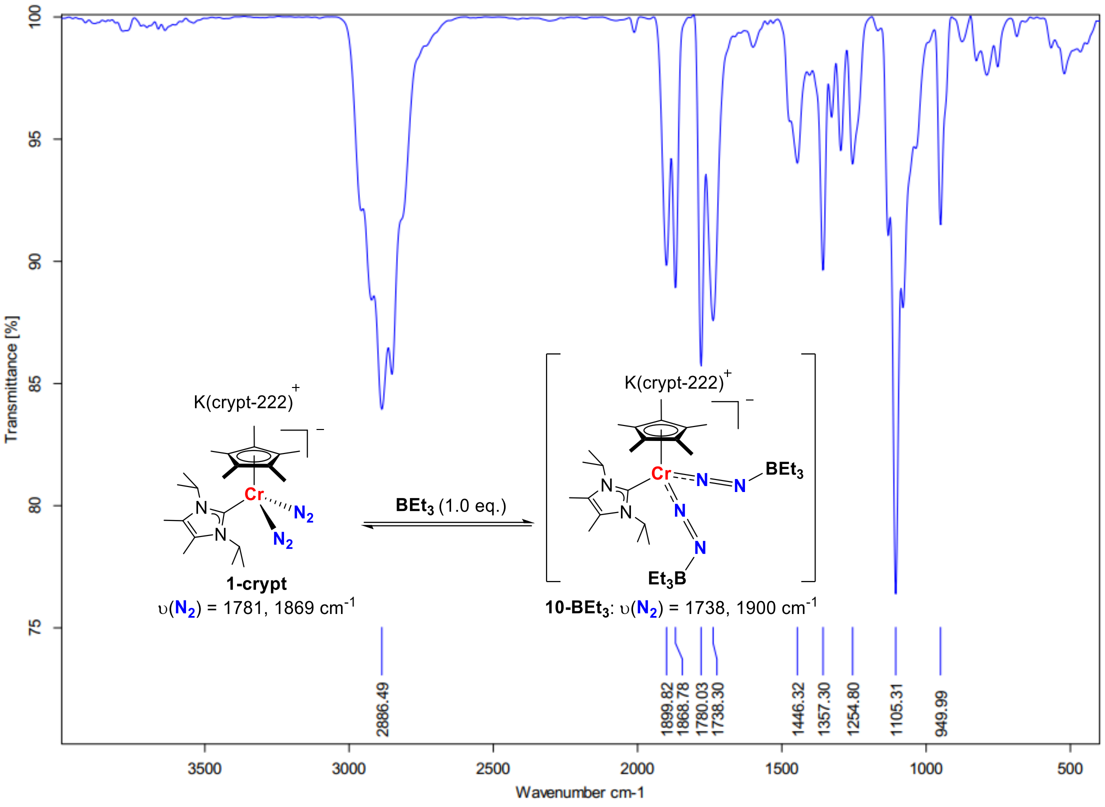

Figure S9 IR spectrum of *in situ* reaction of **1-crypt** and 1.0 equiv BEt_3 in THF/Et_2O at room temperature

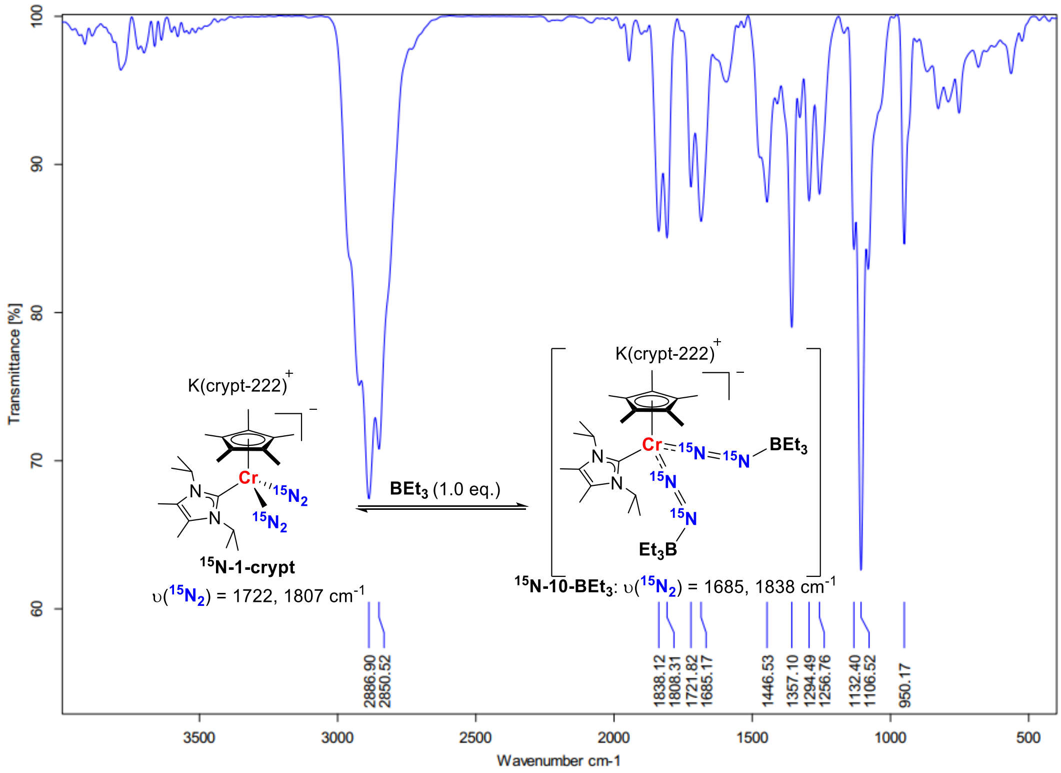

Figure S10 IR spectrum of *in situ* reaction of ^{15}**N-1-crypt** and 1.0 equiv BEt_3 in THF/Et_2O at room temperature

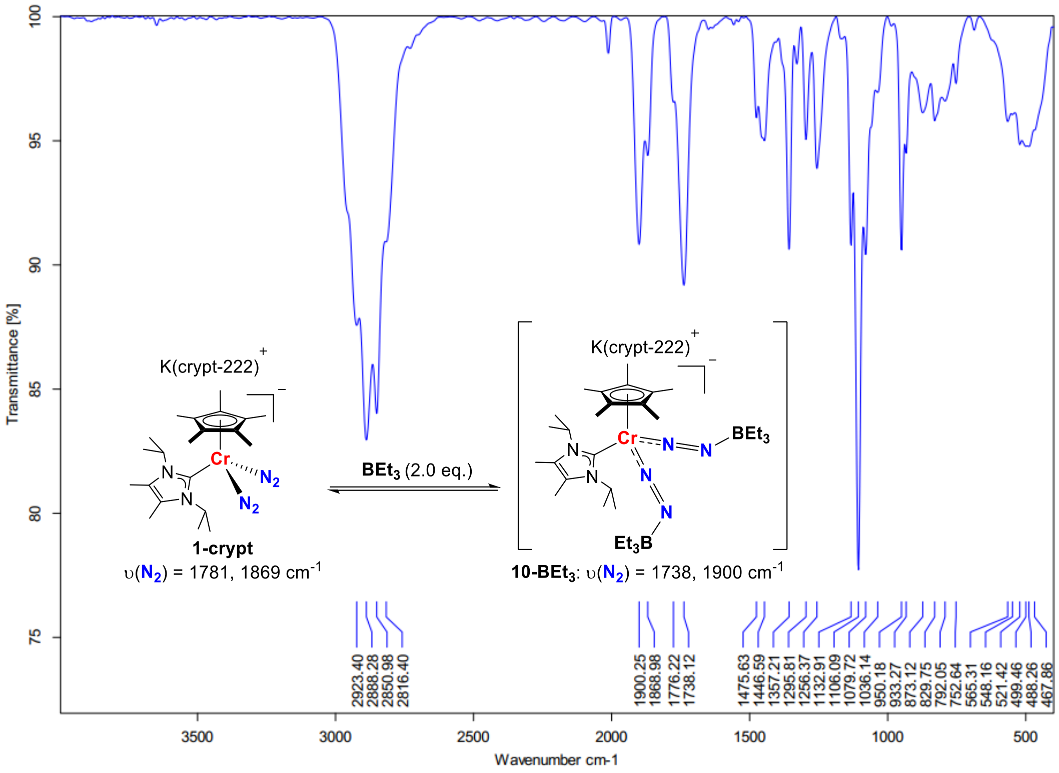

Figure S11 IR spectrum of *in situ* reaction of **1-crypt** and 2.0 equiv BEt_3 in THF/Et_2O at room temperature

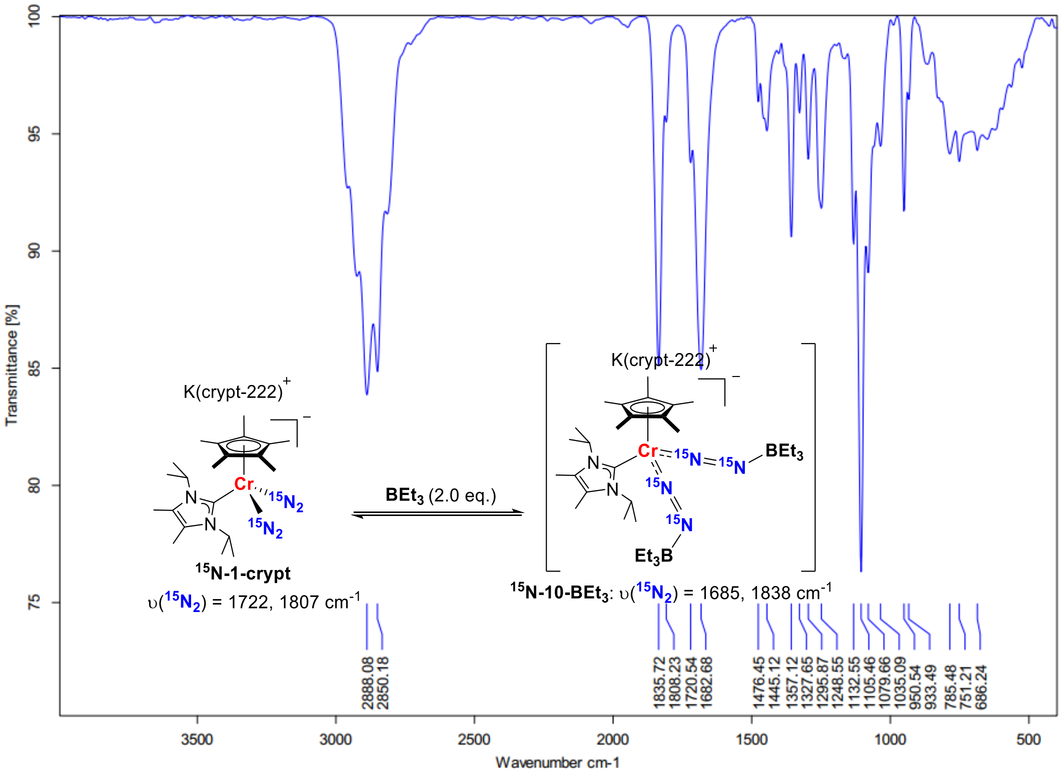

Figure S12 IR spectrum of *in situ* reaction of ^{15}**N-1-crypt** and 2.0 equiv BEt_3 in THF/Et_2O at room temperature

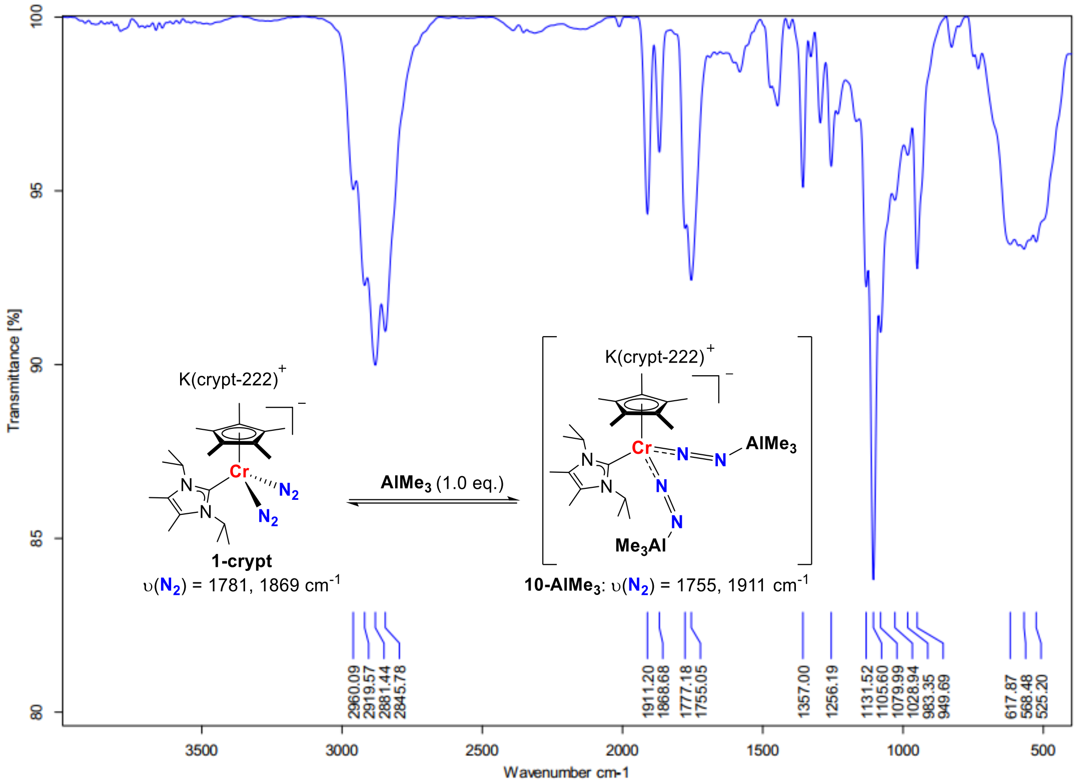

Figure S13 IR spectrum of *in situ* reaction of **1-crypt** and 1.0 equiv $AlMe_3$ in THF/Et_2O at room temperature

4) Copies of NMR Spectra

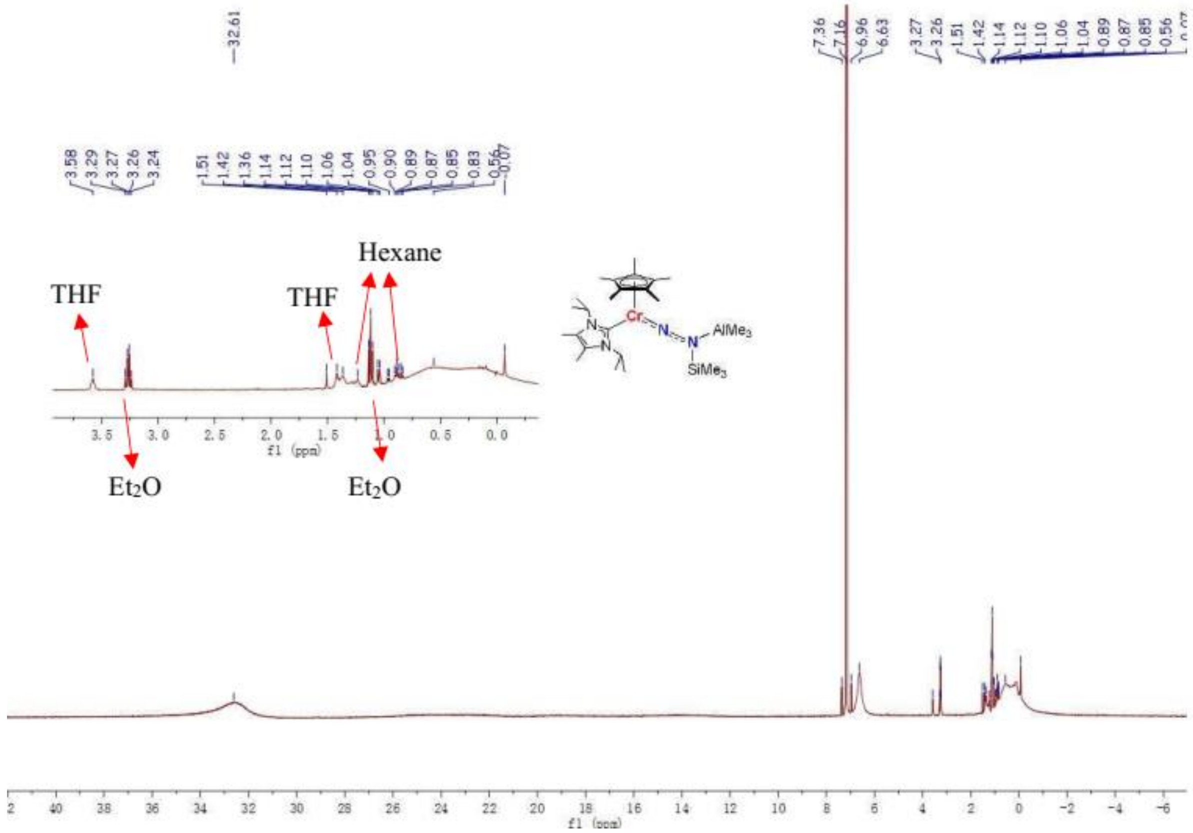

Figure S14 ^{1}H NMR (400 MHz, C_6D_6) spectrum of **2a** at room temperature

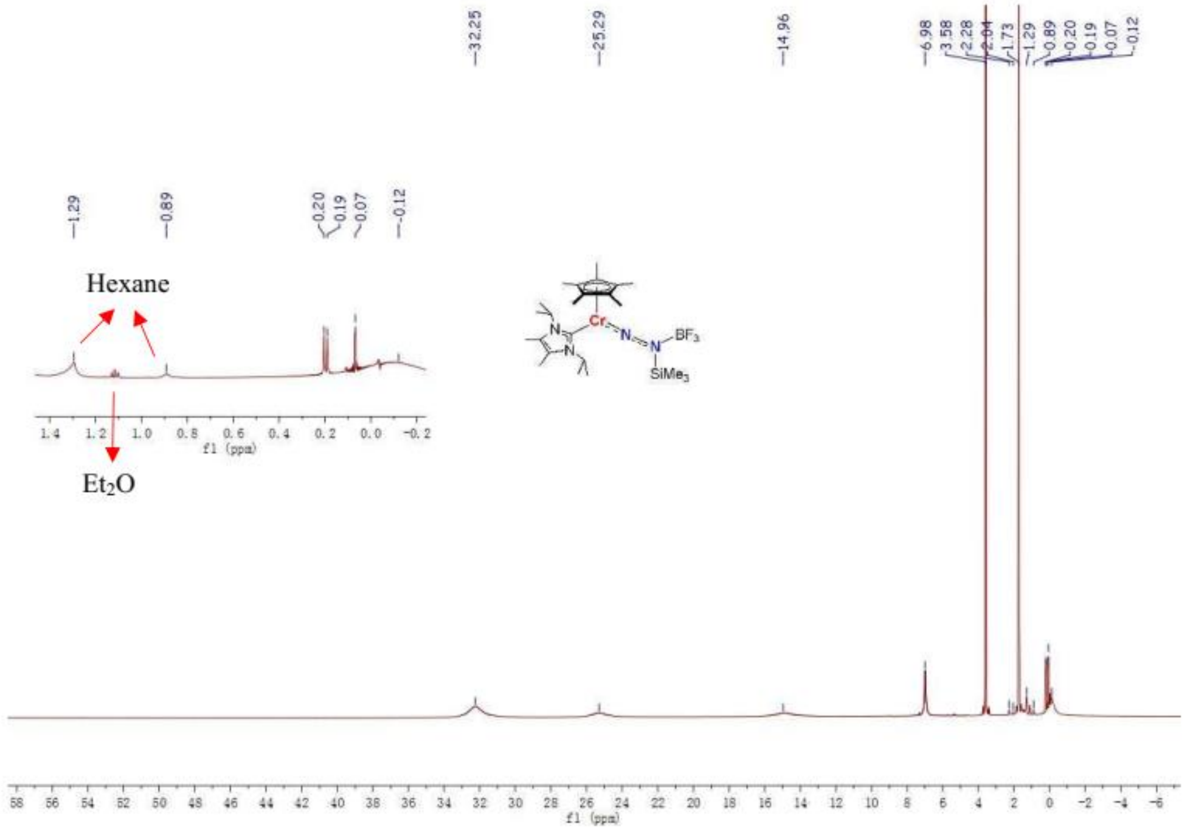

Figure S15 ^{1}H NMR (400 MHz, THF-d^{8}) spectrum of **2b** at room temperature

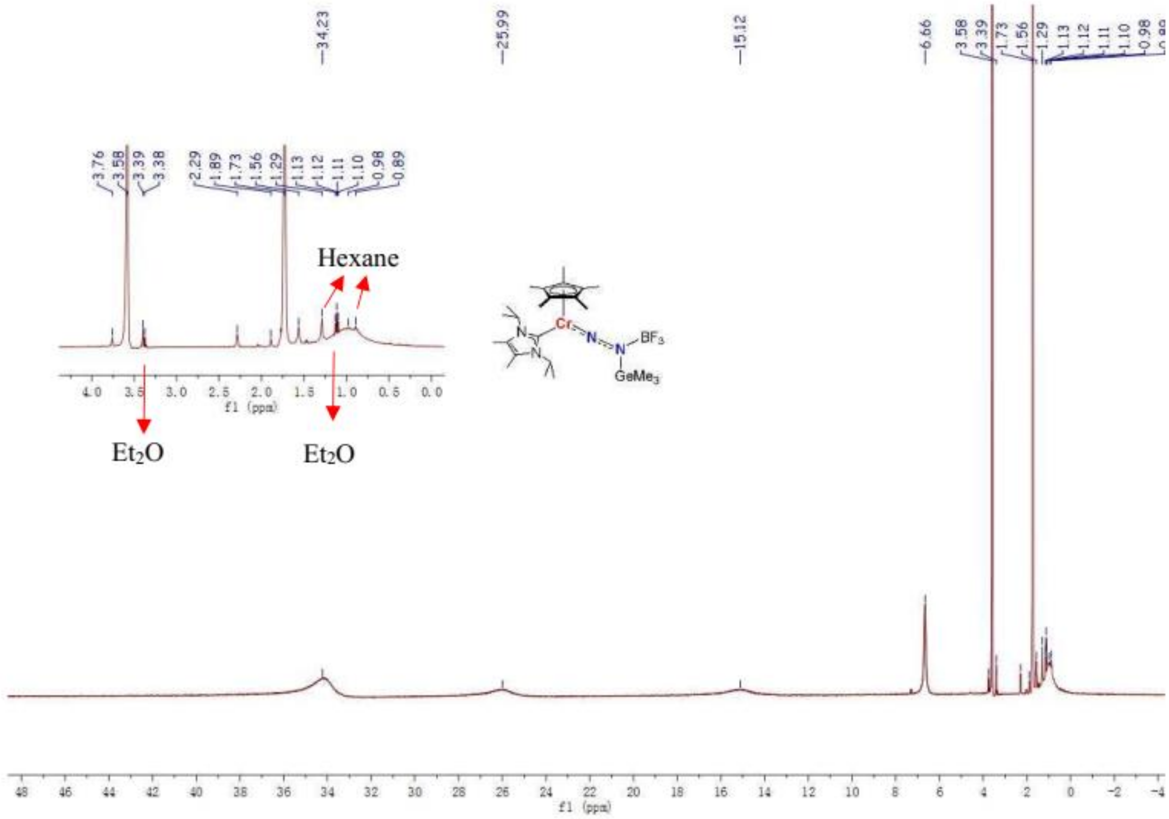

Figure S16 ^{1}H NMR (400 MHz, THF-d^{8}) spectrum of **3** at room temperature

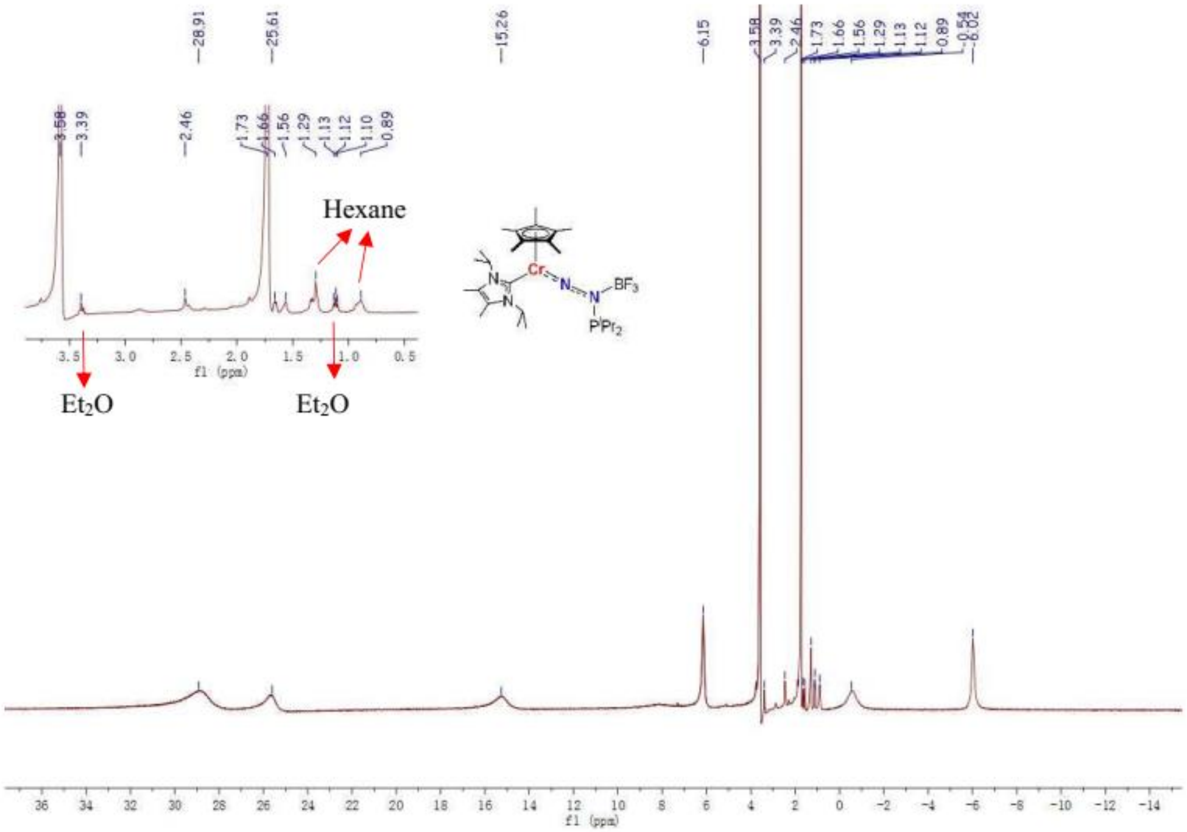

Figure S17 ^{1}H NMR (400 MHz, THF-d^{8}) spectrum of **4** at room temperature

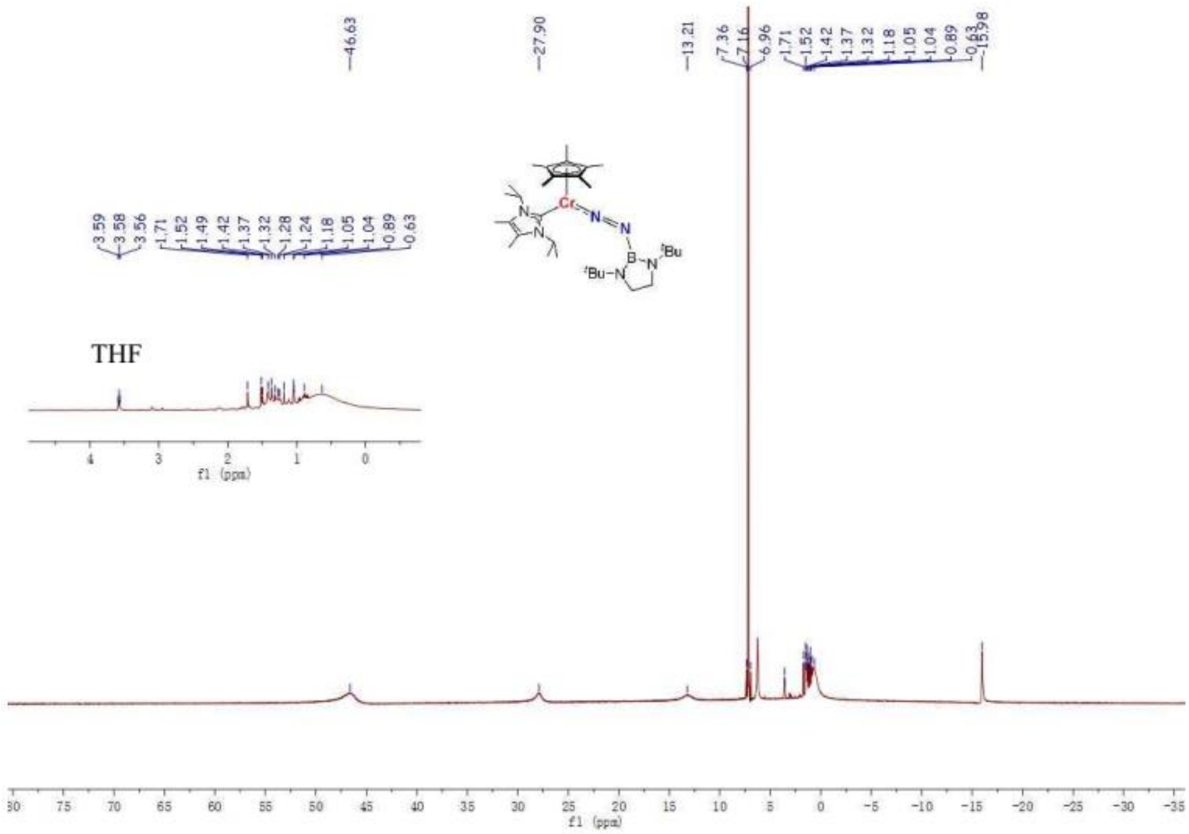

Figure S18 ^{1}H NMR (400 MHz, C_6D_6) spectrum of **6** at room temperature

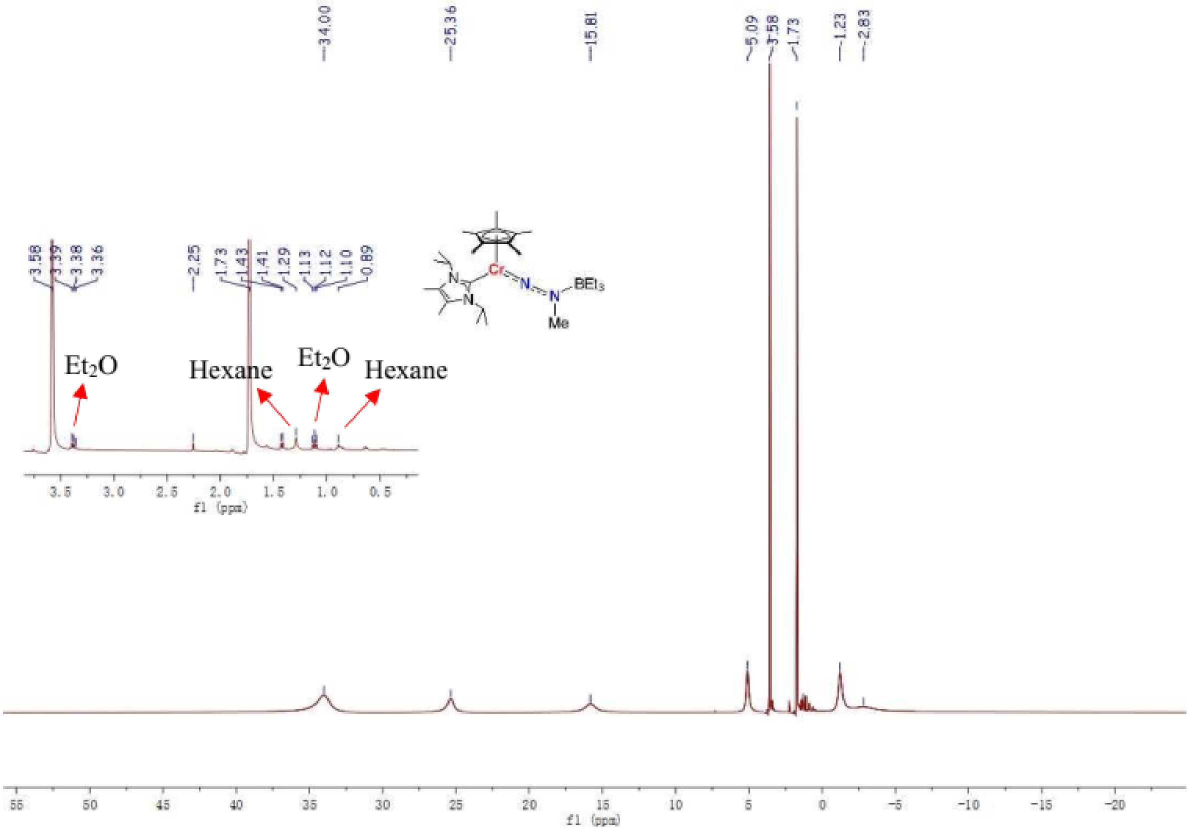

Figure S19 ^{1}H NMR (400 MHz, THF-d^8) spectrum of **8** at room temperature

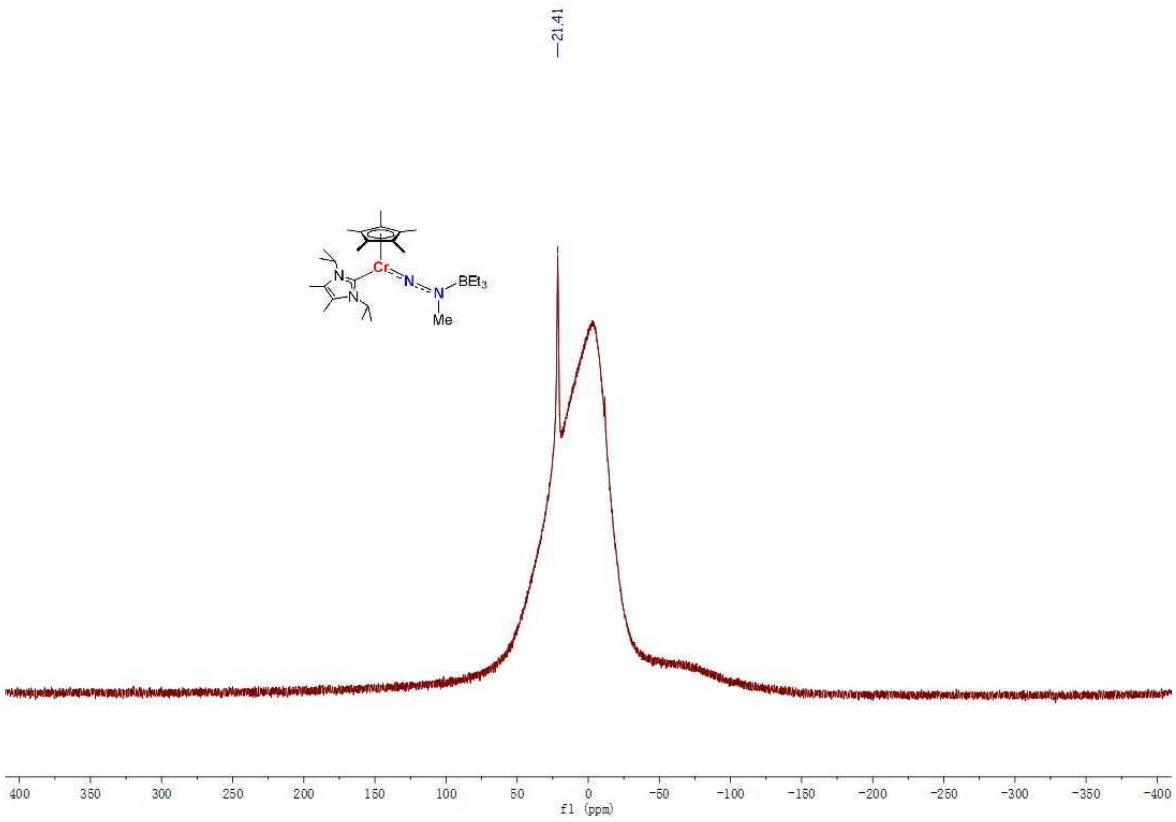

Figure S20 ^{11}B NMR (160 MHz, THF-d^8) spectrum of **8** at room temperature

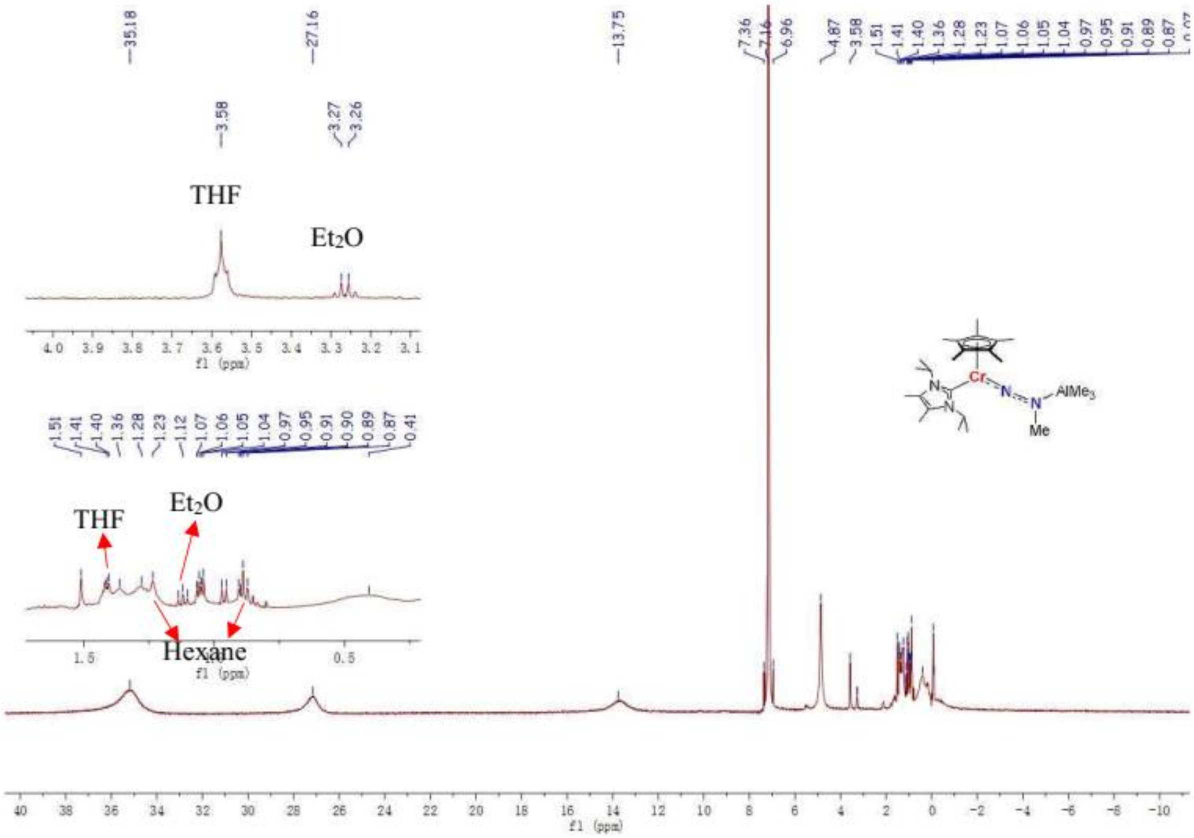

Figure S21 ^{1}H NMR (400 MHz, C_6D_6) spectrum of **9** at room temperature

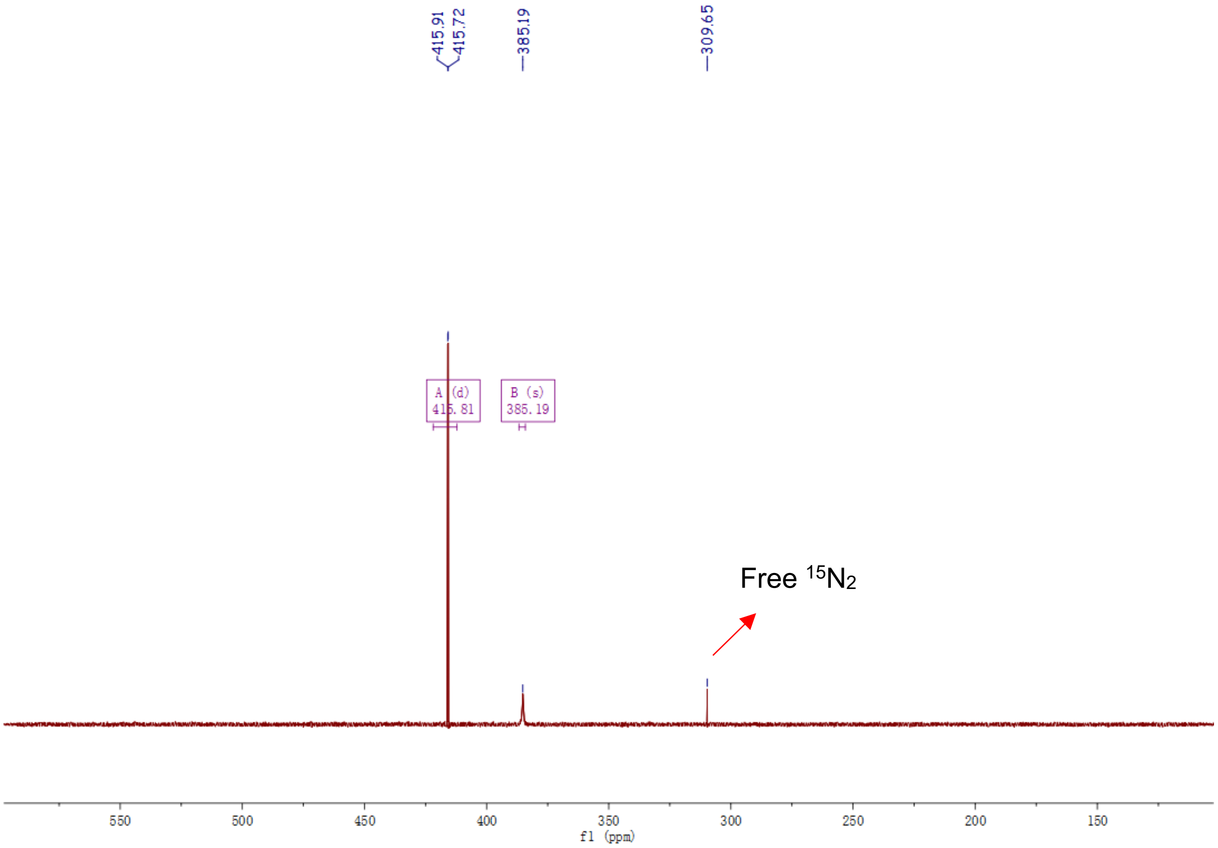

Figure S22 ^{15}N NMR (61 MHz, THF-d^8) spectrum of *in situ* reaction of **1-crypt** and 2.0 equiv. BEt_3 at room temperature

5) Copies of UV-Vis Spectra

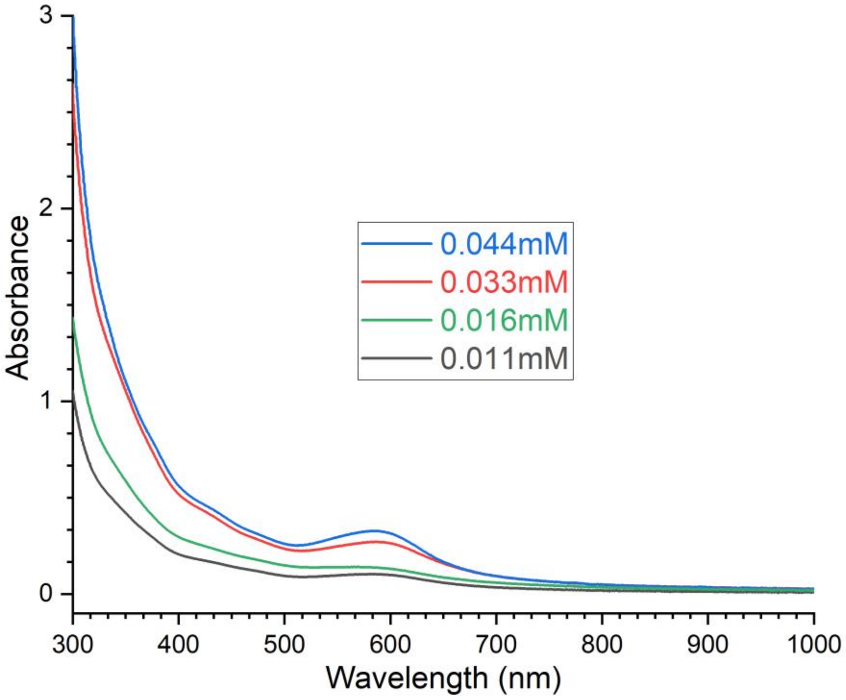

Figure S23 UV-Vis spectra of **2a** in 0.011-0.044 mM solutions in THF at room temperature.

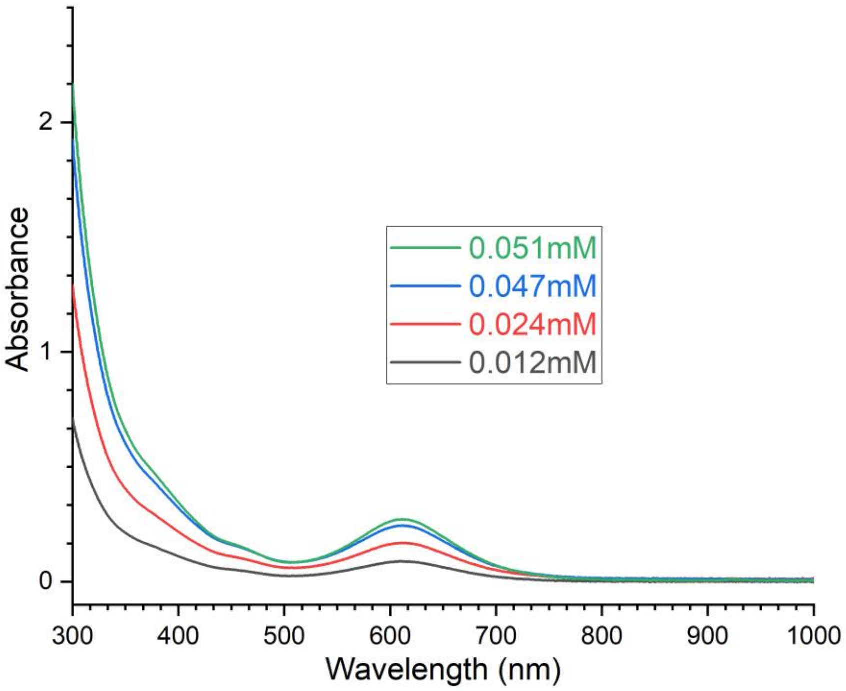

Figure S24 UV-Vis spectra of **2b** in 0.012-0.051 mM solutions in THF at room temperature.

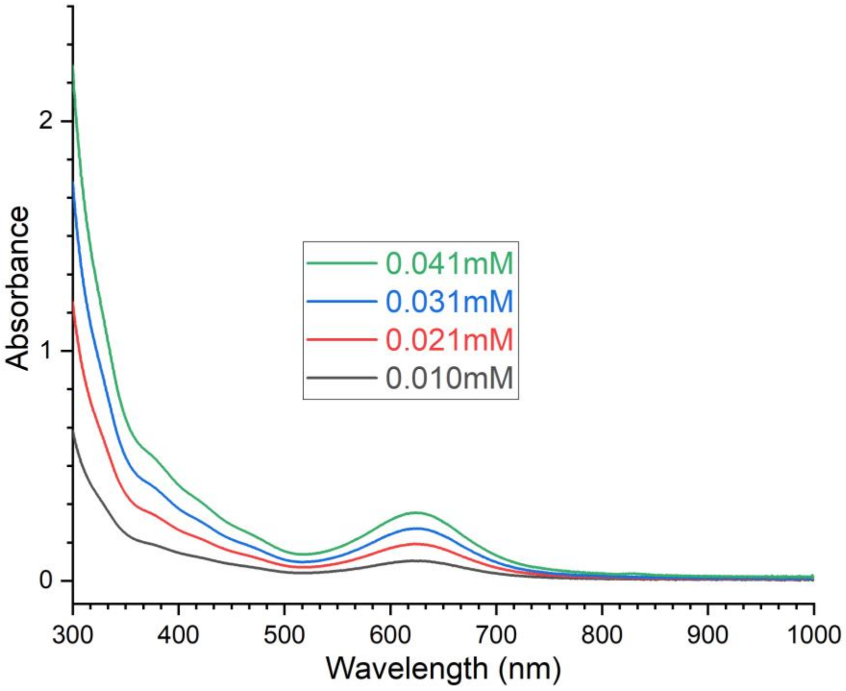

Figure S25 UV-Vis spectra of **3** in 0.010-0.041 mM solutions in THF at room temperature.

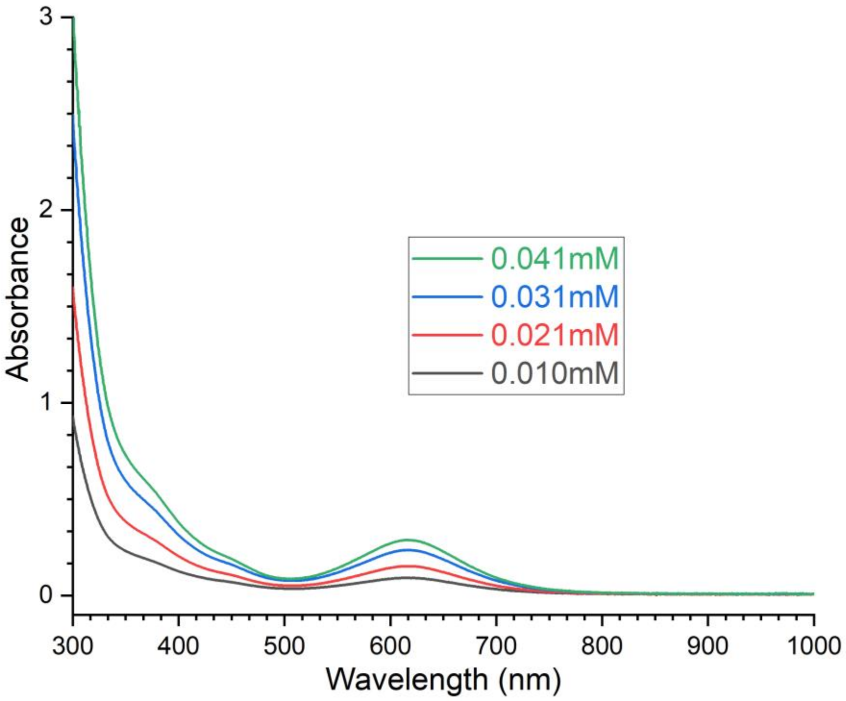

Figure S26 UV-Vis spectra of **4** in 0.010-0.041 mM solutions in THF at room temperature.

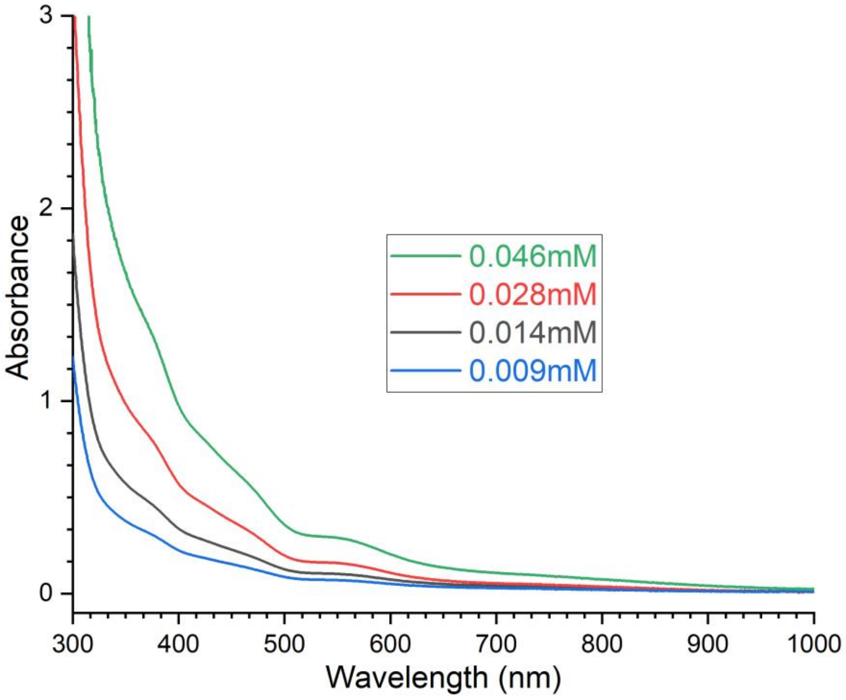

Figure S27 UV-Vis spectra of **6** in 0.009-0.046 mM solutions in THF at room temperature.

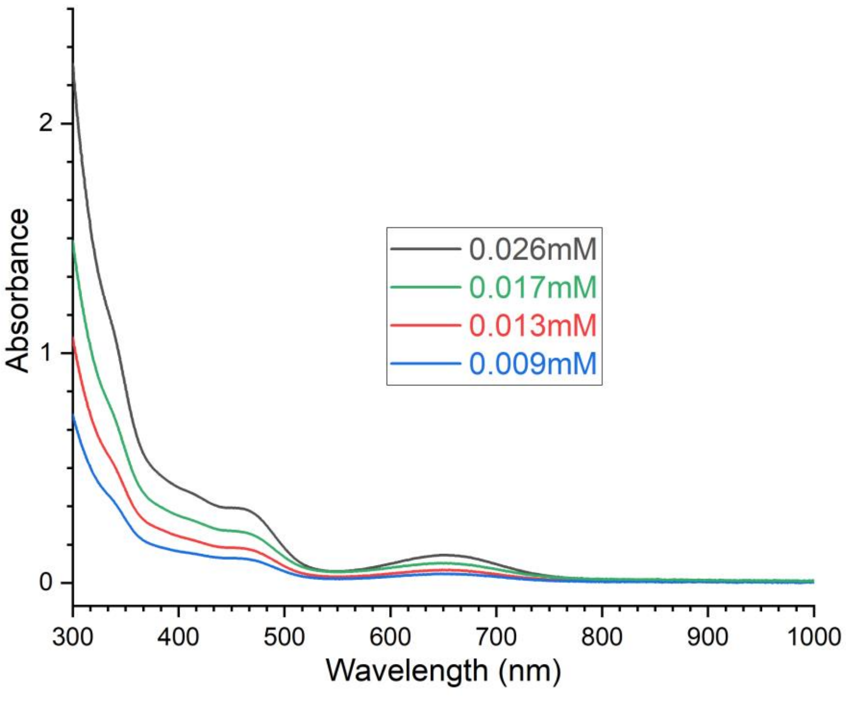

Figure S28 UV-Vis spectra of **8** in 0.009-0.026 mM solutions in THF at room temperature.

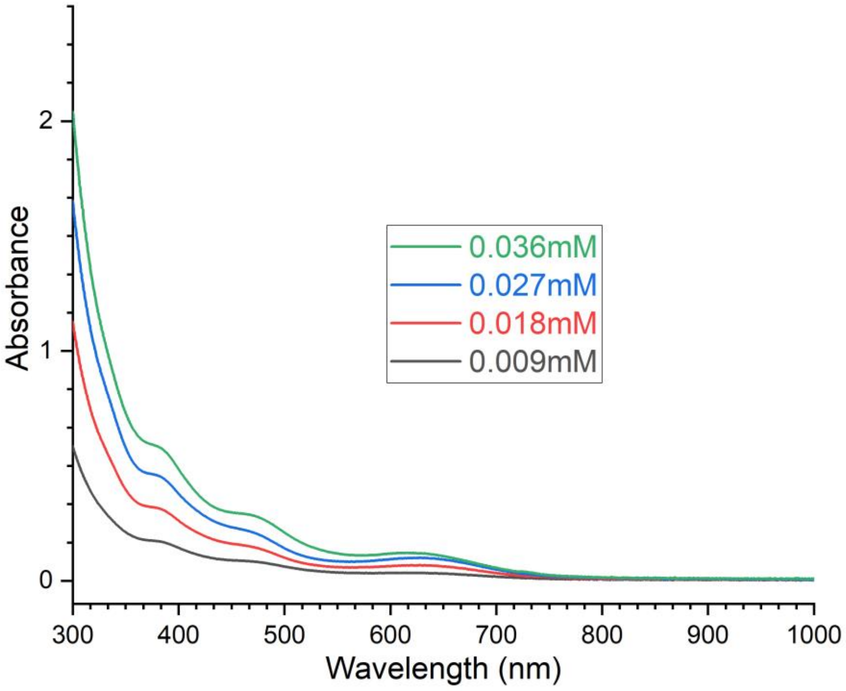

Figure S29 UV-Vis spectra of **9** in 0.009-0.036 mM solutions in THF at room temperature.

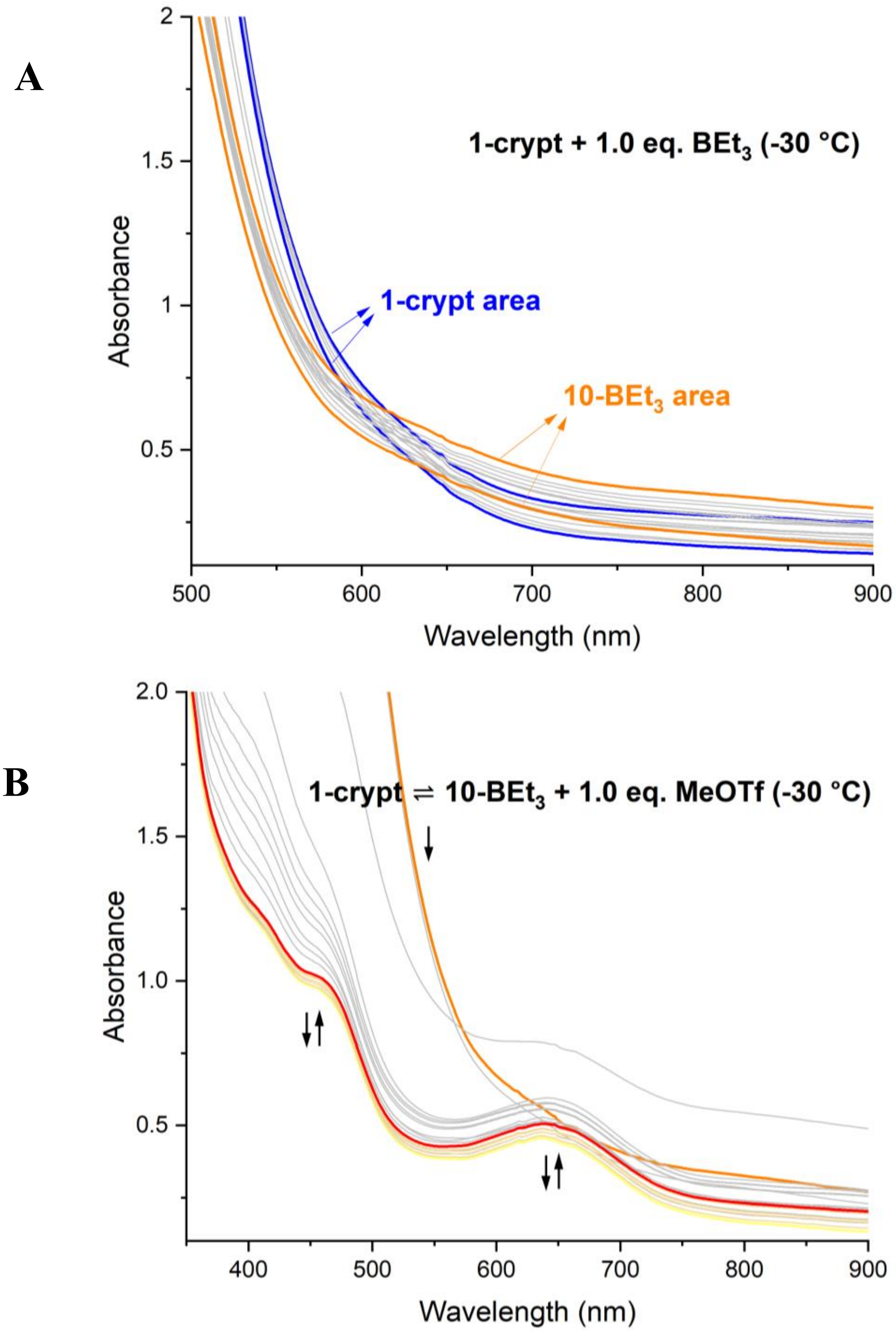

Figure S30 (A) UV-Vis spectral changes of the reaction of Cp*Cr(I^{i}Pr$_2$Me$_2$)(N$_2$)$_2$K(crypt-222) (**1-crypt**) (1.0 mM, topmost blue line) with 1.0 equiv of BEt_3 in THF at -30 °C within about 5 minutes. Following the addition of BEt_3, the spectrum quickly shifts down to the bottommost orange line, then oscillates, moving up and moving down. (B) UV-Vis spectral changes of the reaction of the *in-situ* generated **1-crypt** and **10-BEt$_3$** equilibrium mixture (1.0 mM, orange line) with 1.0 equiv of MeOTf in THF at -30 °C within about 10 minutes (change from orange line to light yellow line to red line).

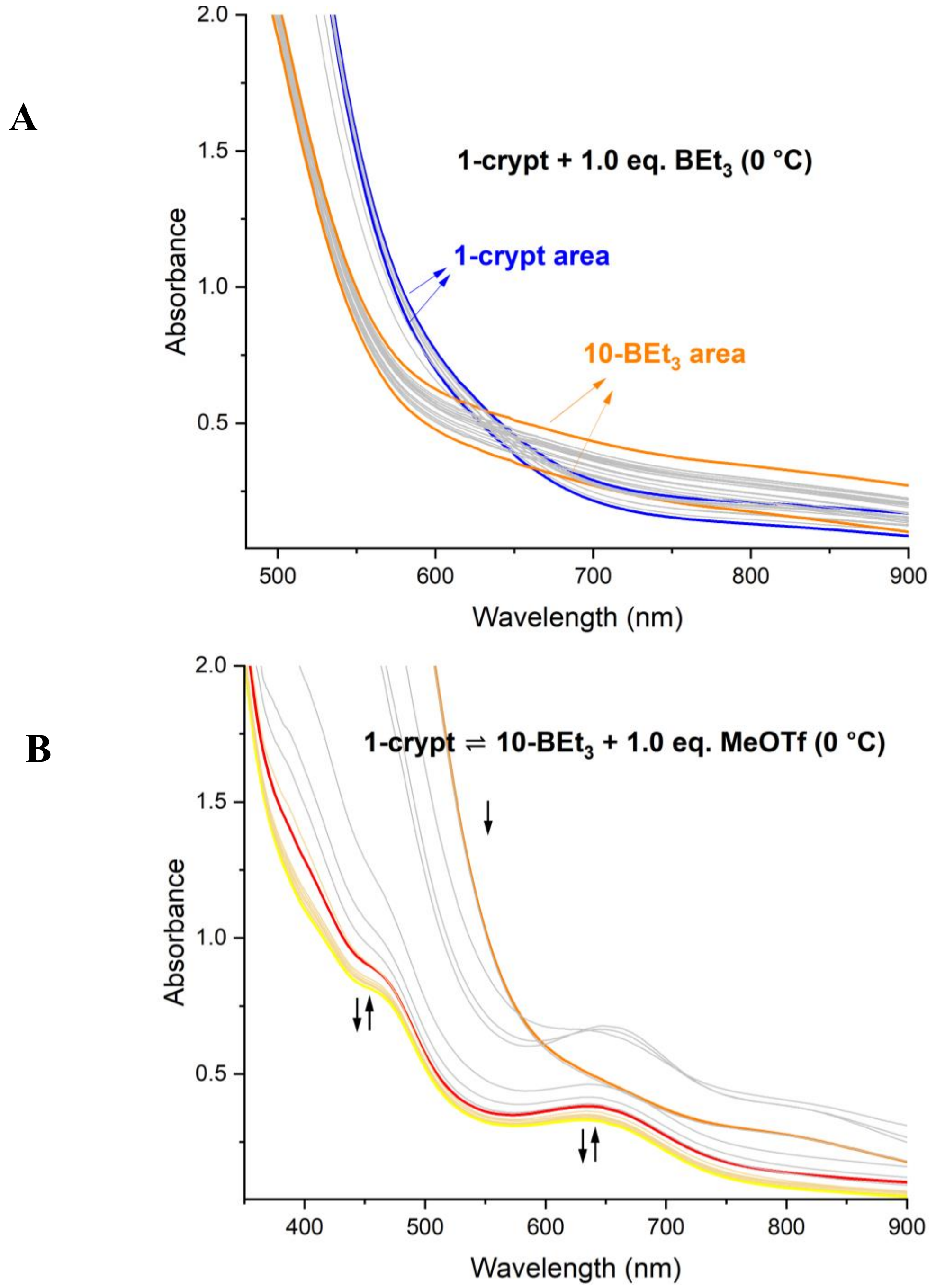

Figure S31 (A) UV-Vis spectral changes of the reaction of $Cp^*Cr(I^iPr_2Me_2)(N_2)_2K$(crypt-222) (**1-crypt**) (1.0 mM, topmost blue line) with 1.0 equiv of BEt_3 in THF at 0 °C within about 5 minutes. Following the addition of BEt_3, the spectrum quickly shifts down to the bottommost orange line, then oscillates, moving up and moving down. (B) UV-Vis spectral changes of the reaction of the *in-situ* generated **1-crypt** and **10-BEt$_3$** equilibrium mixture (1.0 mM, orange line) with 1.0 equiv of MeOTf in THF at 0 °C within about 10 minutes (changes from orange line to light yellow line to red line).

A

B

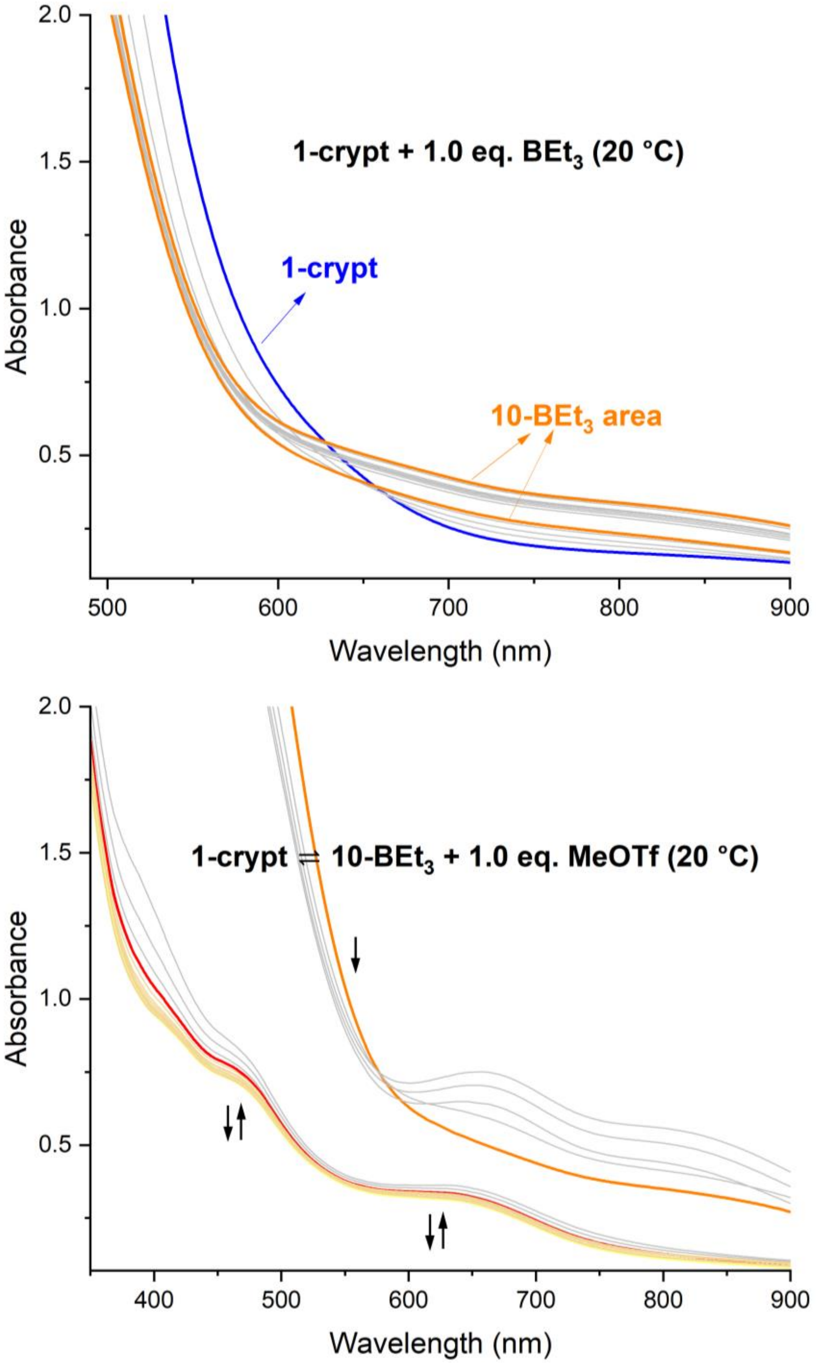

Figure S32 (A) UV-Vis spectral changes of the reaction of $Cp^*Cr(I^iPr_2Me_2)(N_2)_2K$(crypt-222) (**1-crypt**) (1.0 mM, topmost blue line) with 1.0 equiv of BEt_3 in THF at 20 °C within about 5 minutes. Following the addition of BEt_3, the spectrum quickly shifts down to the bottommost orange line, then oscillates, moving up and moving down. (B) UV-Vis spectral changes of the reaction of the *in-situ* generated **1-crypt** and **10-BEt$_3$** equilibrium mixture (1.0 mM, orange line) with 1.0 equiv of MeOTf in THF at 20 °C within about 10 minutes (changes from orange line to light yellow line to red line).

6) X-ray Crystallographic Studies

Data collections were performed at 180 K on Rigaku diffractometer, using monochromated Mo Kα radiation (λ = 0.71073 Å). The structures were solved by SHELXTL or Olex program.[4,5] Refinement was performed on F^2 anisotropically for all the non-hydrogen atoms by the full-matrix least-squares method. The hydrogen atoms were placed at the calculated positions and were included in the structure calculation without further refinement of the parameters. Crystal data, data collection and processing parameters for compounds **2-9** are summarized in following tables. Crystallographic data have been deposited with the Cambridge Crystallographic Data Centre as supplementary publication numbers: CCDC-**2356388 (2a)**, CCDC-**2356389 (2b)**, CCDC-**2356390 (3)**, CCDC-**2356392 (4)**, CCDC-**2356391 (6)**, CCDC-**2356394 (8)**, CCDC-**2356393 (9)**, CCDC-2405607 (**11**). Copies of these data can be obtained free of charge from the Cambridge Crystallographic Data Centre via www.ccdc.cam.ac.uk/data_request/cif.

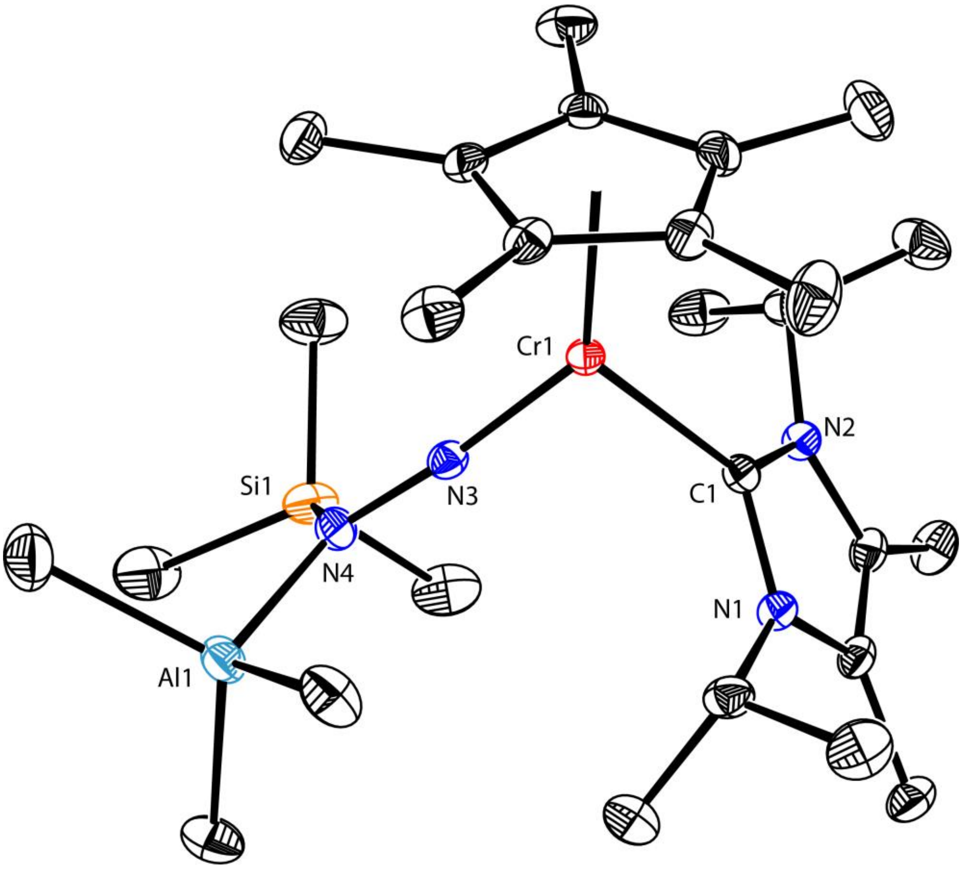

Figure S33 ORTEP drawing of **2a**. Thermal ellipsoids are shown at the 30% probability level. Hydrogen atoms are omitted for clarity. Selected bond lengths (Å) and angles (deg): Cr1-N3 1.6801(3), N3−N4 1.3060(17), N4−Al1 1.9659(17), N3−N4−Si1 111.98(11).

Table S1 X-ray crystallographic data for **2a**

Identification code	**2a**
Empirical formula	$C_{27}H_{53}AlCrN_4Si$
Formula weight	540.80
Temperature/K	180.00(10)
Crystal system	monoclinic
Space group	$P2_1/n$
a/Å	10.9013(5)
b/Å	18.2856(9)
c/Å	15.9641(7)
α/°	90
β/°	90.430(4)
γ/°	90
Volume/$Å^3$	3182.1(3)
Z	4
ρ_{calc} g/cm^3	1.129
μ/mm^{-1}	0.445
F(000)	1176.0
Crystal size/mm^3	0.1 × 0.1 × 0.1
Radiation	MoKα (λ = 0.71073)
2Θ range for data collection/°	5.03 to 54.958
Index ranges	-13 ≤ h ≤ 14, -23 ≤ k ≤ 23, -20 ≤ l ≤ 20
Reflections collected	27175
Independent reflections	7210 [R_{int} = 0.0437, R_{sigma} = 0.0415]
Data/restraints/parameters	7210/0/324
Goodness-of-fit on F^2	1.054
Final R indexes [I>=2σ (I)]	R_1 = 0.0378, wR_2 = 0.0965
Final R indexes [all data]	R_1 = 0.0543, wR_2 = 0.1035
Largest diff. peak/hole / e $Å^{-3}$	0.29/-0.27

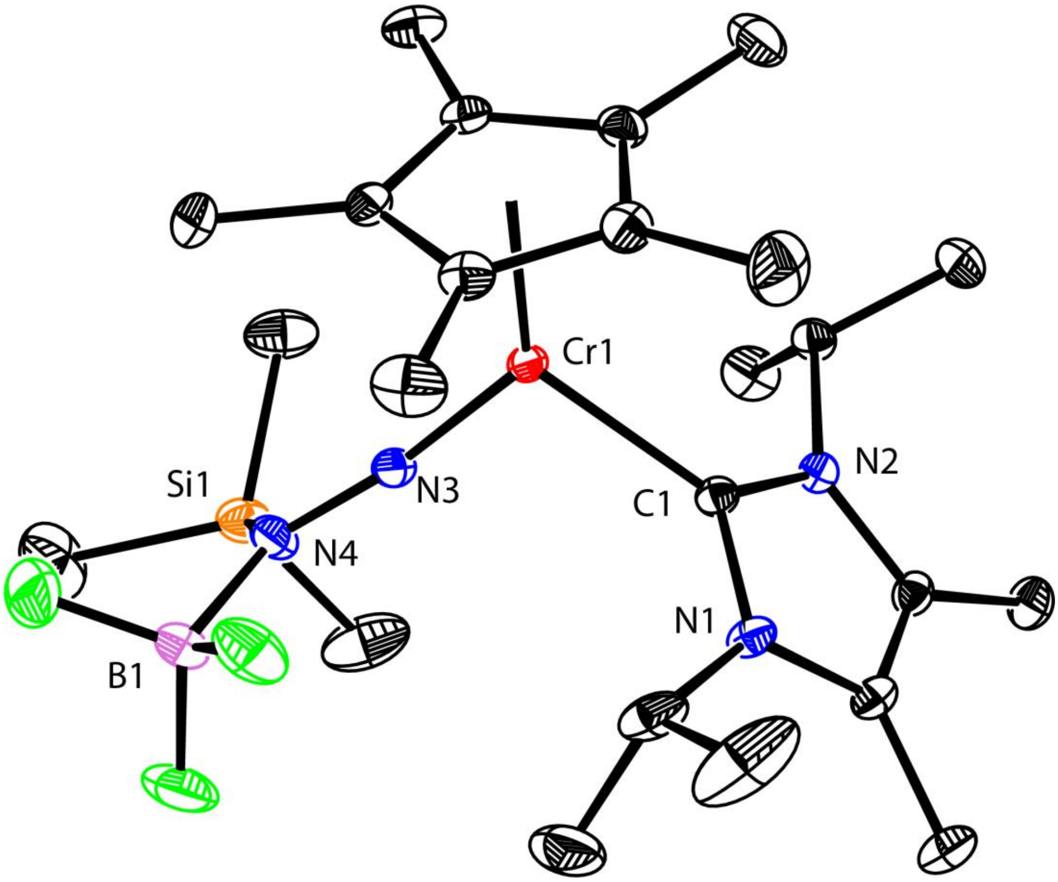

Figure S34 ORTEP drawing of **2b**. Thermal ellipsoids are shown at the 30% probability level. Hydrogen atoms are omitted for clarity. Selected bond lengths (Å) and angles (deg): Cr1-N3 1.6768(13), N3−N4 1.3108(17), N4−B1 1.574(2), N3−N4−Si1 115.34(10).

Table S2 X-ray crystallographic data for **2b**

Identification code	**2b**
Empirical formula	$C_{48}H_{88}B_2Cr_2F_6N_8Si_2$
Formula weight	1073.06
Temperature/K	179.99(10)
Crystal system	monoclinic
Space group	$P2_1/c$
a/Å	17.3984(6)
b/Å	21.1977(5)
c/Å	17.5851(6)
α/°	90
β/°	115.520(4)
γ/°	90
Volume/Å^3	5852.7(4)
Z	4
ρ_{calc} g/cm^3	1.218
μ/mm^{-1}	0.469
F(000)	2288.0
Crystal size/mm^3	0.2 × 0.2 × 0.2
Radiation	MoKα (λ = 0.71073)
2Θ range for data collection/°	4.636 to 54.97
Index ranges	-22 ≤ h ≤ 22, -25 ≤ k ≤ 27, -22 ≤ l ≤ 22
Reflections collected	63064
Independent reflections	13359 [R_{int} = 0.0238, R_{sigma} = 0.0207]
Data/restraints/parameters	13359/0/641
Goodness-of-fit on F^2	1.039
Final R indexes [I>=2σ (I)]	R_1 = 0.0341, wR_2 = 0.0910
Final R indexes [all data]	R_1 = 0.0415, wR_2 = 0.0944
Largest diff. peak/hole / e Å^{-3}	0.41/-0.34

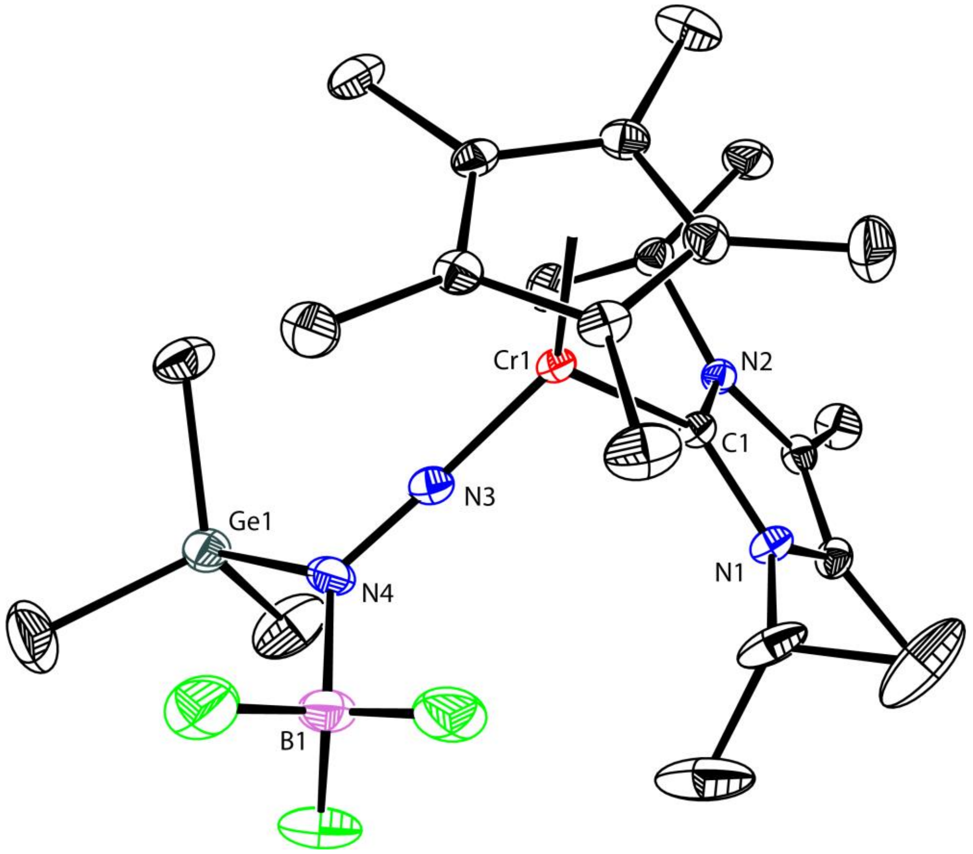

Figure S35 ORTEP drawing of **3**. Thermal ellipsoids are shown at the 30% probability level. Hydrogen atoms are omitted for clarity. Selected bond lengths (Å) and angles (deg): Cr1-N3 1.684(2), N3−N4 1.295(3), N4−B1 1.574(4), N3−N4−Ge1 116.27(18).

Table S3 X-ray crystallographic data for **3**

Identification code	**3**
Empirical formula	$C_{48}H_{88}B_2Cr_2F_6Ge_2N_8$
Formula weight	1162.06
Temperature/K	180.00(10)
Crystal system	monoclinic
Space group	$P2_1/c$
a/Å	17.4734(7)
b/Å	21.2296(7)
c/Å	17.6537(6)
α/°	90
β/°	115.858(5)
γ/°	90
Volume/Å^3	5893.0(4)
Z	4
ρ_{calc} g/cm^3	1.310
μ/mm^{-1}	1.426
F(000)	2432.0
Crystal size/mm^3	0.1 × 0.1 × 0.1
Radiation	MoKα (λ = 0.71073)
2Θ range for data collection/°	3.202 to 54.968
Index ranges	$-16 \leq h \leq 22$, $-20 \leq k \leq 27$, $-22 \leq l \leq 22$
Reflections collected	55918
Independent reflections	13514 [R_{int} = 0.0468, R_{sigma} = 0.0453]
Data/restraints/parameters	13514/0/641
Goodness-of-fit on F^2	1.030
Final R indexes [I>=2σ (I)]	R_1 = 0.0489, wR_2 = 0.1038
Final R indexes [all data]	R_1 = 0.0678, wR_2 = 0.1099
Largest diff. peak/hole / e Å^{-3}	0.76/-0.61

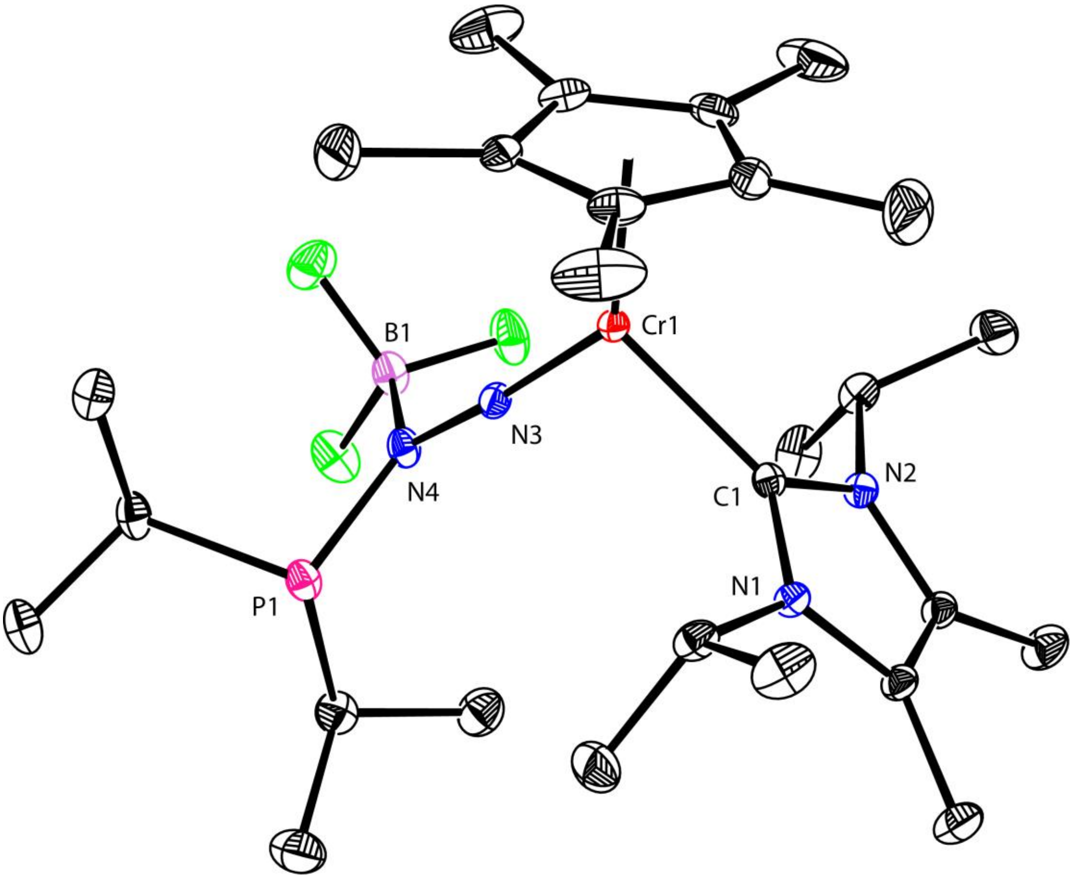

Figure S36 ORTEP drawing of **4**. Thermal ellipsoids are shown at the 30% probability level. Hydrogen atoms are omitted for clarity. Selected bond lengths (Å) and angles (deg): Cr1−N3 1.6753(11), N3−N4 1.3144(16), N4−B1 1.581(2), N3−N4−P1 113.47(10).

Table S4 X-ray crystallographic data for **4**

Identification code	**4**
Empirical formula	$C_{27}H_{49}BCrF_3N_4P$
Formula weight	580.48
Temperature/K	179.99(10)
Crystal system	monoclinic
Space group	$P2_1/n$
a/Å	9.7439(3)
b/Å	18.1925(5)
c/Å	17.8380(5)
α/°	90
β/°	92.607(2)
γ/°	90
Volume/Å^3	3158.80(16)
Z	4
ρ_{calc} g/cm^3	1.221
μ/mm^{-1}	0.452
F(000)	1240.0
Crystal size/mm^3	0.1 × 0.1 × 0.1
Radiation	MoKα (λ = 0.71073)
2Θ range for data collection/°	4.572 to 54.966
Index ranges	$-12 \leq h \leq 12$, $-23 \leq k \leq 23$, $-23 \leq l \leq 20$
Reflections collected	28383
Independent reflections	7228 [R_{int} = 0.0288, R_{sigma} = 0.0295]
Data/restraints/parameters	7228/0/349
Goodness-of-fit on F^2	1.027
Final R indexes [I>=2σ (I)]	R_1 = 0.0335, wR_2 = 0.0839
Final R indexes [all data]	R_1 = 0.0428, wR_2 = 0.0875
Largest diff. peak/hole / e Å^{-3}	0.30/-0.26

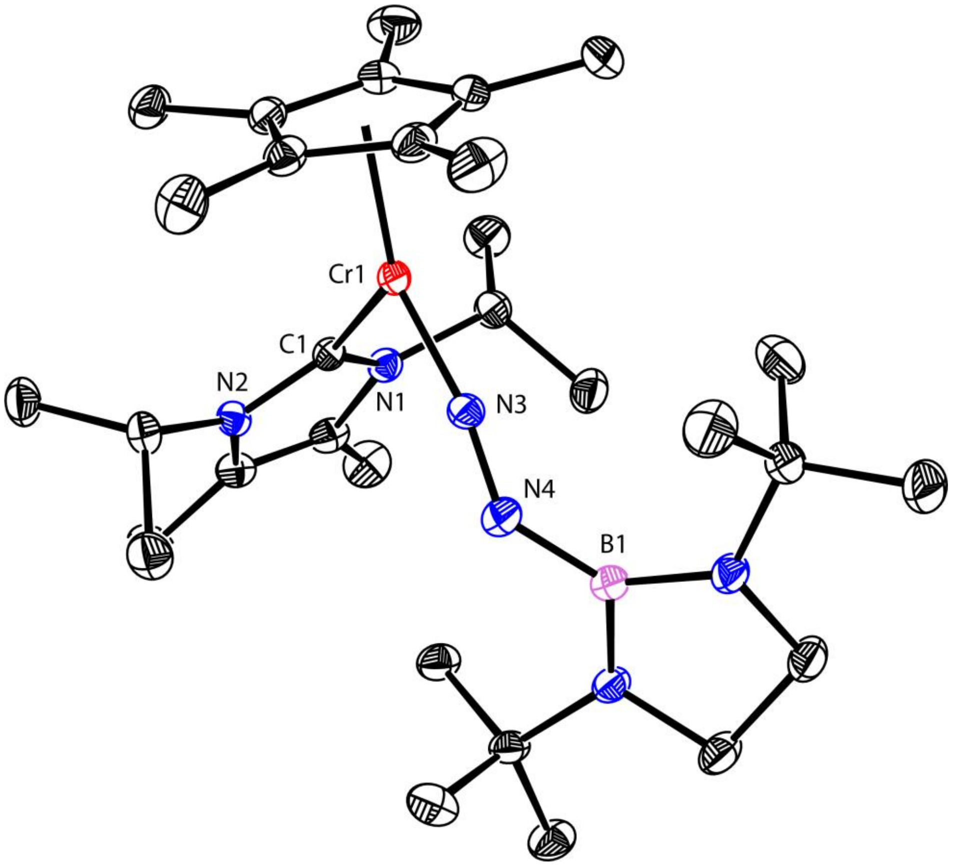

Figure S37 ORTEP drawing of **6**. Thermal ellipsoids are shown at the 30% probability level. Hydrogen atoms are omitted for clarity. Selected bond lengths (Å) and angles (deg): Cr1–N3 1.7104(17), N3–N4 1.231(2), N4–B1 1.423(3), N3–N4–B1 138.56(19).

Table S5 X-ray crystallographic data for **6**

Identification code	**6**
Empirical formula	$C_{31}H_{57}BCrN_6$
Formula weight	576.63
Temperature/K	179.99(10)
Crystal system	orthorhombic
Space group	*Pbca*
a/Å	11.4018(3)
b/Å	20.4671(7)
c/Å	29.1157(9)
α/°	90
β/°	90
γ/°	90
Volume/Å^3	6794.5(4)
Z	8
ρ_{calc} g/cm^3	1.127
μ/mm^{-1}	0.365
F(000)	2512.0
Crystal size/mm^3	0.1 × 0.1 × 0.1
Radiation	MoKα (λ = 0.71073)
2Θ range for data collection/°	3.98 to 54.968
Index ranges	$-13 \le h \le 14$, $-26 \le k \le 23$, $-36 \le l \le 37$
Reflections collected	31756
Independent reflections	7774 [R_{int} = 0.0309, R_{sigma} = 0.0347]
Data/restraints/parameters	7774/0/369
Goodness-of-fit on F^2	1.045
Final R indexes [I>=2σ (I)]	R_1 = 0.0506, wR_2 = 0.1232
Final R indexes [all data]	R_1 = 0.0634, wR_2 = 0.1294
Largest diff. peak/hole / e Å^{-3}	0.82/-0.70

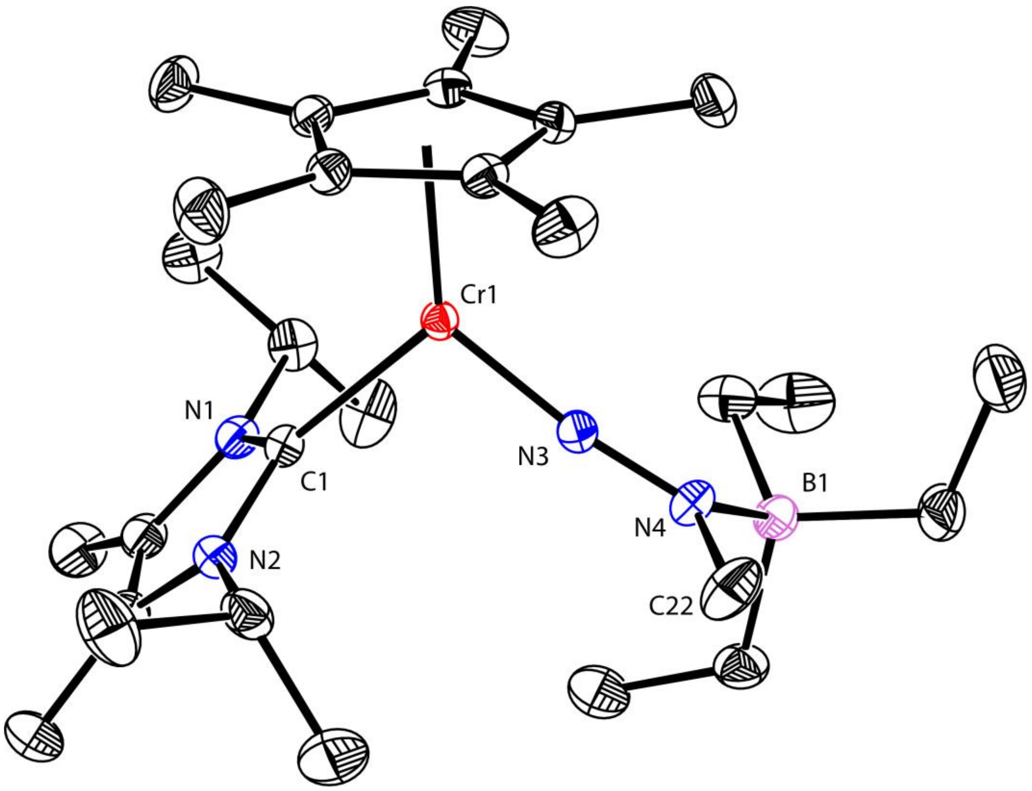

Figure S38 ORTEP drawing of **8**. Thermal ellipsoids are shown at the 30% probability level. Hydrogen atoms are omitted for clarity. Selected bond lengths (Å) and angles (deg): Cr1−N3 1.6902(16), N3−N4 1.289(2), N4−B1 1.618(3), N3−N4−C22 114.19(18).

Table S6 X-ray crystallographic data for **8**

Identification code	**8**
Empirical formula	$C_{28}H_{53}BCrN_4$
Formula weight	508.55
Temperature/K	179.99(10)
Crystal system	orthorhombic
Space group	$P2_12_12_1$
a/Å	10.9154(3)
b/Å	16.1613(4)
c/Å	17.7452(5)
α/°	90
β/°	90
γ/°	90
Volume/Å^3	3130.38(15)
Z	4
ρ_{calc} g/cm^3	1.079
μ/mm^{-1}	0.386
F(000)	1112.0
Crystal size/mm^3	0.1 × 0.1 × 0.1
Radiation	MoKα (λ = 0.71073)
2Θ range for data collection/°	5.042 to 54.966
Index ranges	$-14 \leq h \leq 13$, $-20 \leq k \leq 20$, $-23 \leq l \leq 22$
Reflections collected	20717
Independent reflections	7027 [R_{int} = 0.0266, R_{sigma} = 0.0297]
Data/restraints/parameters	7027/0/322
Goodness-of-fit on F^2	1.043
Final R indexes [I>=2σ (I)]	R_1 = 0.0297, wR_2 = 0.0767
Final R indexes [all data]	R_1 = 0.0329, wR_2 = 0.0779
Largest diff. peak/hole / e Å^{-3}	0.20/-0.17
Flack parameter	-0.001(6)

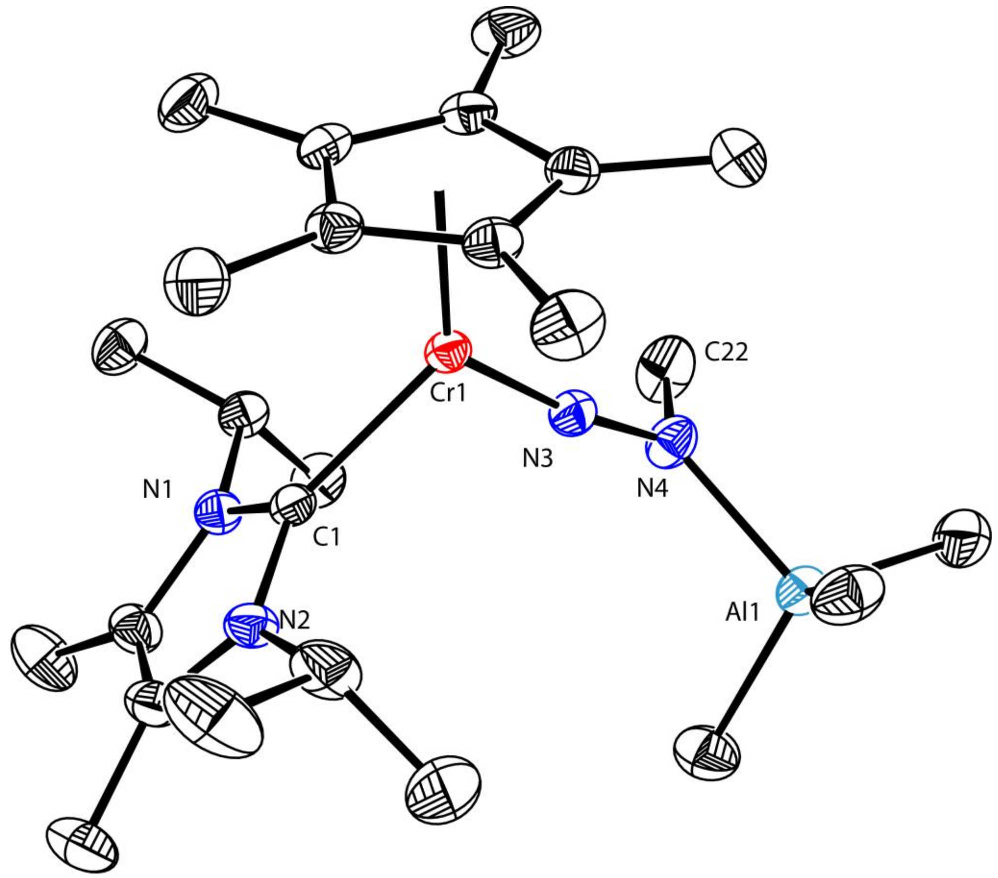

Figure S39 ORTEP drawing of **9**. Thermal ellipsoids are shown at the 30% probability level. Hydrogen atoms are omitted for clarity. Selected bond lengths (Å) and angles (deg): Cr1-N3 1.694(2), N3−N4 1.286(3), N4−Al1 1.944(2), N3−N4−Al1 121.28(16).

Table S7 X-ray crystallographic data for **9**

Identification code	**9**
Empirical formula	$C_{25.22}H_{47.44}AlCrN_4$
Formula weight	485.73
Temperature/K	179.99(10)
Crystal system	monoclinic
Space group	$P2_1/n$
a/Å	10.7000(3)
b/Å	16.3665(4)
c/Å	17.0052(4)
α/°	90
β/°	101.290(2)
γ/°	90
Volume/Å^3	2920.35(13)
Z	4
ρ_{calc} g/cm^3	1.105
μ/mm^{-1}	0.439
F(000)	1055.0
Crystal size/mm^3	0.1 × 0.1 × 0.1
Radiation	MoKα (λ = 0.71073)
2Θ range for data collection/°	4.162 to 54.97
Index ranges	-13 ≤ h ≤ 13, -21 ≤ k ≤ 21, -22 ≤ l ≤ 22
Reflections collected	23527
Independent reflections	6675 [R_{int} = 0.0280, R_{sigma} = 0.0332]
Data/restraints/parameters	6675/37/315
Goodness-of-fit on F^2	1.020
Final R indexes [I>=2σ (I)]	R_1 = 0.0440, wR_2 = 0.1177
Final R indexes [all data]	R_1 = 0.0604, wR_2 = 0.1261
Largest diff. peak/hole / e Å^{-3}	0.43/-0.23

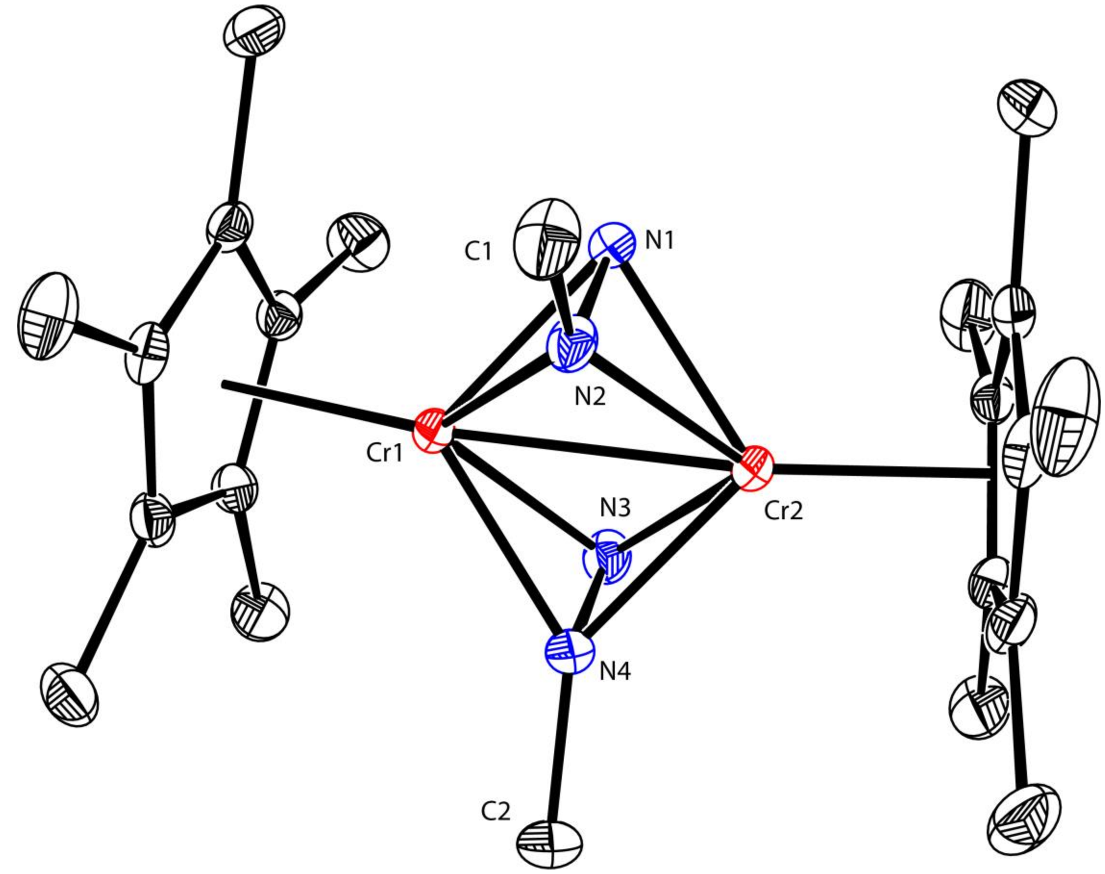

Figure S40 ORTEP drawing of **11**. Thermal ellipsoids are shown at the 30% probability level. Hydrogen atoms are omitted for clarity. Selected bond lengths (Å) and angles (deg): Cr1−N1 1.9613(15), Cr1−N2 1.9545(14), Cr1−N3 1.9549(14), Cr1−N4 1.9520(14), Cr2−N1 1.9562(15), Cr2−N2 1.9524(14), Cr1−N3 1.9609(14), Cr1−N4 1.9547(14), N1−N2 1.389(2), N3−N4 1.387(2), Cr1−Cr2 2.4348(4); N1−N2−C1 119.39(15), N3−N4−C2 119.42(17)

Table S8 X-ray crystallographic data for **11**

Identification code	**11**
Empirical formula	$C_{22}H_{36}Cr_2N_4$
Formula weight	460.55
Temperature/K	180.00(14)
Crystal system	triclinic
Space group	*P-1*
a/Å	8.6431(4)
b/Å	11.1879(7)
c/Å	13.9240(8)
α/°	66.730(5)
β/°	80.509(4)
γ/°	69.244(5)
Volume/Å^3	1156.14(12)
Z	2
ρ_{calc} g/cm^3	1.323
μ/mm^{-1}	0.954
F(000)	488.0
Crystal size/mm^3	0.1 × 0.1 × 0.1
Radiation	MoKα (λ = 0.71073)
2Θ range for data collection/°	4.232 to 54.968
Index ranges	$-9 \le h \le 11$, $-14 \le k \le 14$, $-15 \le l \le 18$
Reflections collected	17492
Independent reflections	5298 [R_{int} = 0.0286, R_{sigma} = 0.0328]
Data/restraints/parameters	5298/0/265
Goodness-of-fit on F^2	1.048
Final R indexes [I>=2σ (I)]	R_1 = 0.0311, wR_2 = 0.0877
Final R indexes [all data]	R_1 = 0.0368, wR_2 = 0.0910
Largest diff. peak/hole / e Å^{-3}	0.35/-0.35

7) Computational Details

Density functional theory (DFT) calculations were performed using ORCA 6.0.0 to investigate the electronic structures[6]. All geometric structures were optimized at the TPSSh/ def-TZVP level of theory[7], incorporating dispersion corrections via the Becke-Johnson damping scheme (D3BJ)[8]. The optimized geometries closely match the single-crystal structures, supporting the validity of the computational approach. Additionally, to further ensure accuracy, we conducted single-point energy calculations on the optimized geometries using the double-hybrid functional PWPB95 with def2-QZVPP basis sets[9]. Solvent effects were considered by employing the SMD implicit solvent model with tetrahydrofuran (THF) as the solvent in these single-point calculations[10]. We have carefully validated the spin states of all paramagnetic species, ensuring that the computed electronic structures are physically meaningful. The figures of wavefunction analysis were prepared by Visual Molecular Dynamics (VMD) program and Multiwfn[11]. The optimized geometries were provided in the supplementary information as .xyz files. UV-Vis spectrum of **2b** was computed using the long-range-corrected DFT functional CAM-B3LYP[12] with the def2-TZVP basis sets based on the optimized geometric structure using the Gaussian 16 package[13]. For the ΔG calculations, we used single-point energies from PWPB95/def2-QZVPP combined with vibrational analysis results from TPSSh/def-TZVP at a reaction temperature of -30 °C. To better deal with low frequencies, we applied a quasi-RRHO treatment, with entropy interpolation between the harmonic oscillator and free-rotor approximations using *Shermo* program[14]. For the calculated IR analysis, based on the *Database of Frequency Scale Factors for Electronic Model Chemistries*, we applied a frequency scaling factor of 0.96[15].

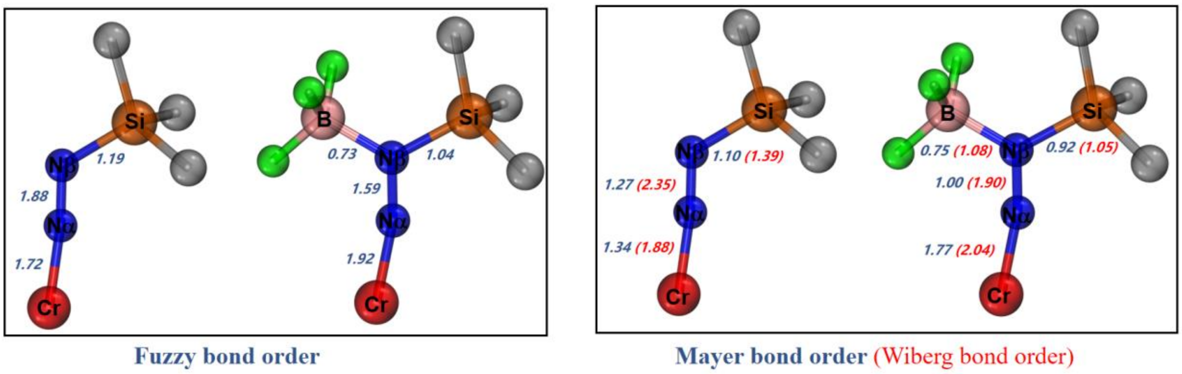

Figure S41 Comparison of Fuzzy bond order, Mayer bond order and Wiberg bond order for [Cp*(I^{i}Pr$_2$Me$_2$)Cr(NNSiMe$_3$)] (left) and **2b** (right). Wavefunctions were generated using the PWPB95/def2-QZVPP level of theory.

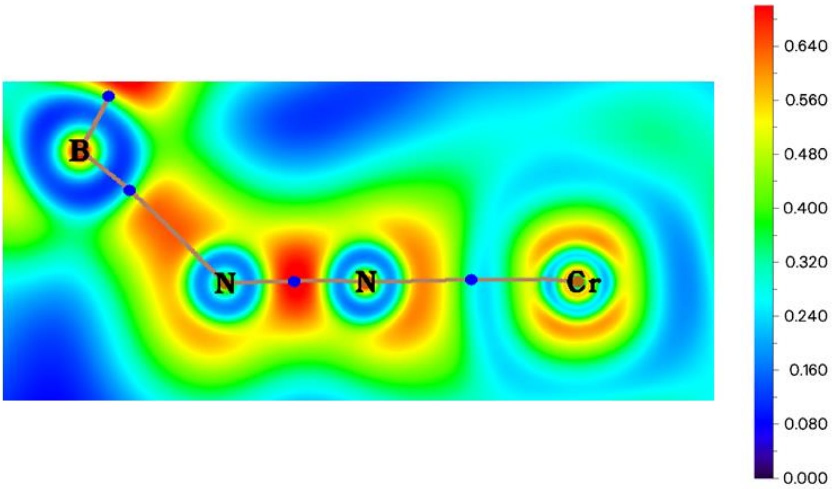

Figure S42 The plot of localized orbital locator (LOL) map of the **10-BEt₃**. (Notes: This analysis identified (3, -1) bond critical points (BCPs), marked as blue dots. The low electron density values at the B–N (3, -1) BCP, along with positive Laplacian values ($\nabla^2\rho(r)$), indicate that the B–N interactions are dative bonds. The localized orbital locator (LOL) map, which provides a real-space visualization of electron (de)localization, providing a real-space visualization of electron (de)localization, further illustrates the dative bond nature of the B–N bond.) Wavefunctions were generated using the PWPB95/def2-QZVPP level of theory.

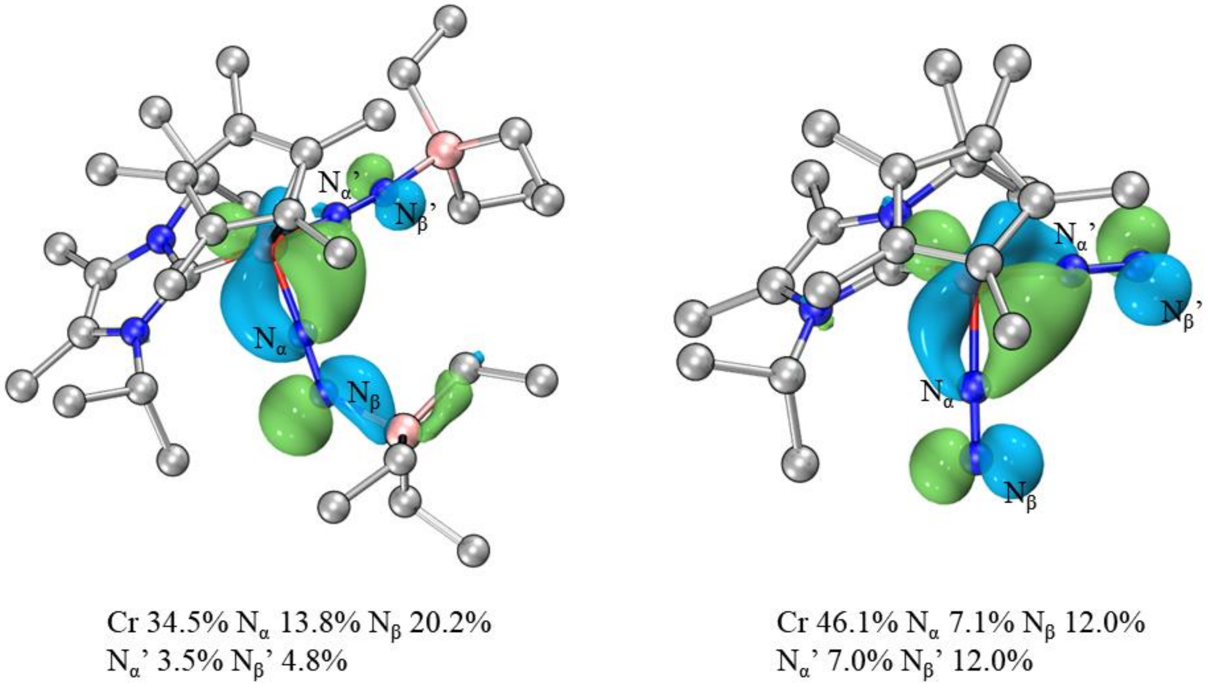

Figure S43 HOMO of anion part of **10-BEt₃** (left) and **1-crypt** (right). Contributions were determined using wavefunctions generated by the double-hybrid functional PWPB95/def2-QZVPP and analyzed through the Hirshfeld method, as implemented in Multiwfn.

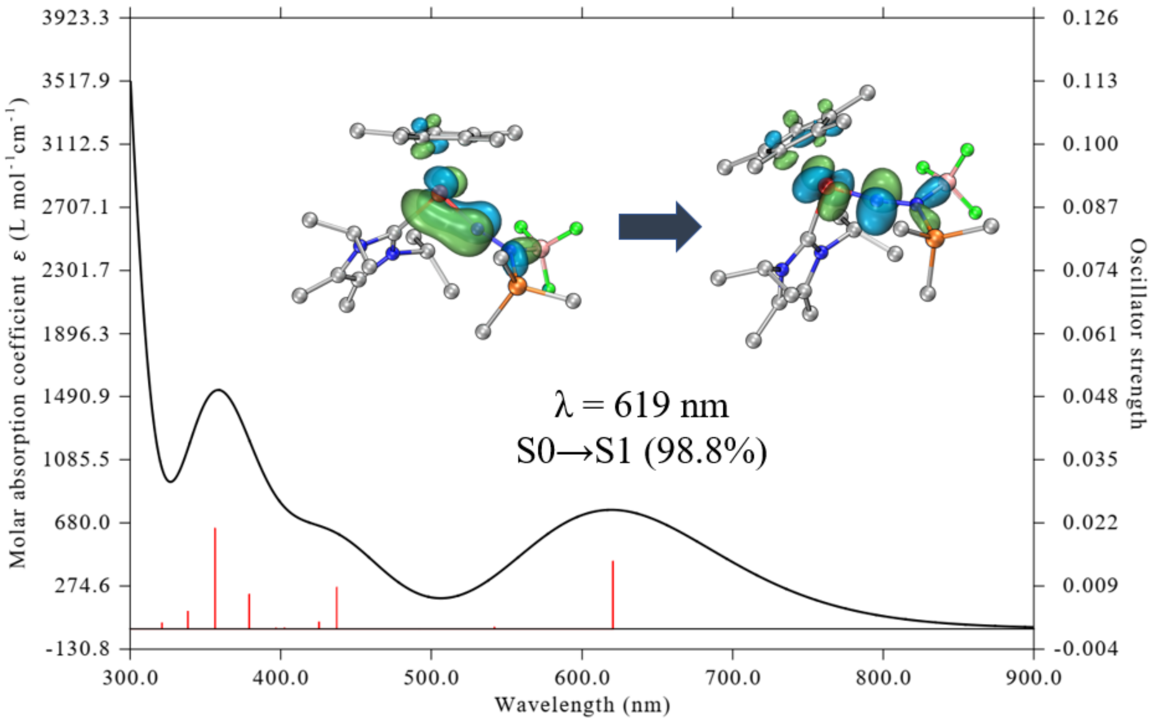

Figure S44 UV-Vis spectrum of **2b** computed using the long-range-corrected DFT functional CAM-B3LYP with the def2-TZVP basis set in a TD-DFT calculation. (Notes: Solvent effects were incorporated by applying the SMD implicit solvent model with THF as the solvent. In the computed UV-vis spectrum, the characteristic peak around 620 nm corresponds to the S0→S1 transition. NTO analysis revealed that this absorption is primarily dominated by the π to π* excitation within the Cr=N=N unit.)

Table S9 Calculated energies (in Hartree). The solvent model was applied during the single-point energy calculations. ***Note:*** The default geometry optimization includes THF as a solvent using the SMD model. However, for complex **2b**, there is always a small imaginary frequency when the solvent model is applied during the optimization, despite several attempts to eliminate it. Therefore, for complex **2b** and the corresponding thermodynamic data calculations, the geometry optimization was performed without the solvent model.

	G (TPSSh/def-TZVP)	G-E (TPSSh/def-TZVP)	Single point energy (PWPB95/def2-QZVPP)	G corrected by *Shermo* at -30 °C
**geometry optimized without solvent model**				
BF_3	-324.698941	-0.013159	-324.593301	-324.600382
MeOTf	-1001.552152	0.031234	-1001.376399	-1001.338315
BF_3•Et_2O	-558.363659	0.115721	-558.226877	-558.102941
Et_2O	-233.659946	0.106346	-233.601615	-233.488626
Cp*(I^iPr_2Me_2)Cr(NNSiMe_3)	-2494.208773	0.571054	-2493.772434	-2493.194048
**2b**	-2818.918886	0.583241	-2818.439346	-2817.847603
**geometry optimized with THF as the solvent**				
BEt_3	-262.505823	0.163990	-262.430888	-262.262105
MeOTf	-1001.560774	0.030993	-1001.376860	-1001.338994
**1-crypt** anion	-2457.127109	0.666885	-2456.696831	-2193.762070
TS **1-crypt** anion with MeOTf	-3196.150357	0.528566	-3195.609204	-3195.071290
1-crypt coordinate 1.0 eq. BEt_3	-2457.127109	0.666885	-2456.696831	-2456.023038
TS **1-crypt** coordinate 1.0 eq. BEt_3 with MeOTf	-3458.658763	0.722694	-3458.058249	-3457.326439
**1-crypt** coordinate 2.0 eq. BEt_3	-2719.624872	0.857715	-2719.152250	-2718.287510
TS **1-crypt** coordinate 2.0 eq. BEt_3 with MeOTf	-3721.156986	0.913362	-3720.523690	-3719.601183

8) References

(1) (a) D. F. Evans, 400. The Determination of the Paramagnetic Susceptibility of Substances in Solution by Nuclear Magnetic Resonance. *J. Chem. Soc.* **1959**, 2003-2005. (b) S. K. Sur, Measurement of Magnetic Susceptibility and Magnetic Moment of Paramagnetic Molecules in Solution by High-Field Fourier Transform NMR Spectroscopy. *J. Magn. Reson.* **1989**, *82*, 169-173.

(2) Z.-B. Yin, B. Wu, G.-X. Wang, J. Wei, Z. Xi, Dinitrogen Functionalization Affording Chromium Diazenido and Side-on η^2-hydrazido Complexes. *J. Am. Chem. Soc.* **2023**, *145*, 7065-7070.

(3) L. Weber, I. Domke, W. Greschner, K. Miqueu, A. Chrostowska, P. Baylère, Synthetic, Cyclovoltammetric, and UV-Photoelectron Spectroscopic Studies of 2,3-Dihydro-1H-1,3,2-diazaboroles and 1,3,2-Diazaborolidines. *Organometallics* **2005**, *24*, 5455-5463.

(4) G. M. Sheldrick, SHELXTL 5.10 for Windows NT: *Structure Determination Software Programs*; Bruker Analytical X-ray Systems, Inc.: Madison, WI (1997).

(5) O. V. Dolomanov, L. J. Bourhis, R. J. Gildea, J. A. K. Howard, H. Puschmann, *OLEX2:* a complete structure solution, refinement and analysis program. *J. Appl. Cryst.* **2009**, *42*, 339-341.

(6) F. Neese, Software update: The ORCA program system—Version 5.0. *WIREs Comput. Mol. Sci.* **12,** e1606 (2022).

(7) J. P. Perdew, J. Tao, V. N. Staroverov, G. E. Scuseria, Meta-generalized gradient approximation: Explanation of a realistic nonempirical density functional. *J. Chem. Phys.* **2004**, *120*, 6898-6911.

(8) S. Grimme, J. Antony, S. Ehrlich, H. Krieg, A consistent and accurate ab initio parametrization of density functional dispersion correction (DFT-D) for the 94 elements H-Pu. *J. Chem. Phys.* **2010**, *132*, 154104.

(9) L. Goerigk, S. Grimme, Efficient and Accurate Double-Hybrid-Meta-GGA Density Functionals-Evaluation with the Extended GMTKN30 Database for General Main Group Thermochemistry, Kinetics, and Noncovalent Interactions. *J. Chem. Theory Comput.* **2011**, *7*, 291–309.

(10) A. V. Marenich, C. J. Cramer, D. G. Truhlar, Universal Solvation Model Based on Solute Electron Density and on a Continuum Model of the Solvent Defined by the Bulk Dielectric Constant and Atomic Surface Tensions. *J. Phys. Chem. B* **2009**, *113*, 6378–6396.

(11) (a) W. Humphrey, A. Dalke, K. Schulten, VMD: Visual molecular dynamics. *J. Mol.*

Graph. **1996**, *14*, 33–38. (b) T. Lu, F. Chen, *J. Comput. Chem.* **2012**, *33*, 580-592.

(12) T. Yanai, D. P. Tew, N. C. Handy, A new hybrid exchange–correlation functional using the Coulomb-attenuating method (CAM-B3LYP). *Chem. Phys. Lett.* **2004**, *393*, 51-57.

(13) Frisch, M. J.; Trucks, G. W.; Schlegel, H. B.; Scuseria, G. E.; Robb, M. A.; Cheeseman, S109 J. R.; Scalmani, G.; Barone, V.; Petersson, G. A.; Nakatsuji, H.; Li, X.; Caricato, M.; Marenich, A.V.; Bloino, J.; Janesko, B. G.; Gomperts, R.; Mennucci, B.; Hratchian, H. P.; Ortiz, J. V.; Izmaylov, A. F.; Sonnenberg, J. L.; Williams-Young, D.; Ding, F.; Lipparini, F.; Egidi, F.; Goings, J.; Peng, B.; Petrone, A.; Henderson, T.; Ranasinghe, D.; Zakrzewski, V. G.; Gao, J.; Rega, N.; Zheng, G.; Liang, W.; Hada, M.; Ehara, M.; Toyota, K.; Fukuda, R.; Hasegawa, J.; Ishida, M.; Nakajima, T.; Honda, Y.; Kitao, O.; Nakai, H.; Vreven, T.; Throssell, K; Montgomery, J. A., Jr.; Peralta, J. E.; Ogliaro, F.; Bearpark, M. J.; Heyd, J. J.; Brothers, E. N.; Kudin, K. N.; Staroverov, V. N.; Keith, T. A.; Kobayashi, R.; Normand, J.; Raghavachari, K.; Rendell, A. P.; Burant, J. C.; Iyengar, S. S.; Tomasi, J.; Cossi, M.; Millam, J. M.; Klene, M.; Adamo, C.; Cammi, R.; Ochterski, J. W.; Martin, R. L.; Morokuma, K.; Farkas, O.; Foresman, J. B.; Fox, D. J. Gaussian 16, Revision C.01, Gaussian, Inc., Wallingford CT, 2019.

(14) T. Lu, Q. Chen, Shermo: A general code for calculating molecular thermochemistry properties. *Comput. Theor. Chem.* **2021**, *1200*, 113249.

(15) I. M. Alecu, J. Zheng, Y. Zhao, D. G. Truhlar, Computational Thermochemistry: Scale Factor Databases and Scale Factors for Vibrational Frequencies Obtained from Electronic Model Chemistries. *J. Chem. Theory Comput.* **2010**, *6*, 2872-2887.
